# Transcriptomic Evidence of a Link between Cell Wall Biogenesis, Pathogenesis, and Vigor in Walnut Root and Trunk Diseases

**DOI:** 10.3390/ijms25020931

**Published:** 2024-01-11

**Authors:** Houston J. Saxe, Sriema L. Walawage, Bipin Balan, Charles A. Leslie, Patrick J. Brown, Gregory T. Browne, Daniel A. Kluepfel, Andreas Westphal, Abhaya M. Dandekar

**Affiliations:** 1 Department of Plant Sciences, University of California, Davis, CA 95616, USA; hsaxe@ucdavis.edu (H.J.S.); slwalawage@ucdavis.edu (S.L.W.); caleslie@ucdavis.edu (C.A.L.); pjbrown@ucdavis.edu (P.J.B.); 2 United States Department of Agriculture’s Agricultural Research Service Crops Pathology and Genetics Research Unit, Department of Plant Pathology, University of California, Davis, CA 95616, USA; gregory.browne@usda.gov (G.T.B.); dakluepfel@ucdavis.edu (D.A.K.); 3 Department of Nematology, University of California, Riverside, CA 92521, USA; andreas.westphal@ucr.edu

**Keywords:** RNA-seq, functional genomics, plant bioinformatics, disease resistance, plant growth, trait discovery, plant–pathogen interaction

## Abstract

Crown gall disease (*Agrobacterium tumefaciens*), crown/root rot disease (*Phytophthora* spp.), root lesion disease (*Pratylenchus vulnus*) and tree vigor are key traits affecting the productivity and quality of walnuts in California. Unchallenged hybrid rootstocks were analyzed by RNA-seq to examine pre-formed factors affecting these traits. Enrichment analysis of the differentially expressed genes revealed that the increased expression of cell wall biogenesis-related genes plays a key role in susceptibility to *A. tumefaciens*, susceptibility to *Phytophthora* spp. and increased vigor. Analysis of the predicted subcellular loci of the encoded proteins revealed that many gene products associated with vigor and susceptibility were targeted to the plasma membrane and extracellular space, connecting these traits to sustaining barrier function. We observed that RNA processing and splicing, along with predicted nuclear targeting, were associated with resistance to *A. tumefaciens*, resistance to *Phytophthora* spp. and low vigor. Four genes within the *J. microcarpa* QTL region for resistance to *A. tumefaciens* and *Phytophthora* spp. were represented among our transcripts, with two of the genes being differentially expressed in association with resistance to *A. tumefaciens* and decreased vigor. No differential expression related to *Phytophthora* spp. or *P. vulnus* resistance was observed in this region. Additionally, the *J. microcarpa* haplotype expressed more transcripts associated with resistance to *A. tumefaciens*, *Phytophthora* spp. and low vigor, but not *P. vulnus*, than the *J. regia* haplotype. We also report unique and shared hormone and defense responses associated with each trait. This research suggests a link between cell wall biogenesis, vigor and critical root diseases of walnut.

## 1. Introduction

The sustainable production of English walnuts (*Juglans regia*) is important for California’s agriculture and rural economy. In 2020, walnuts ranked 11th in California commodities, with an estimated value of USD 957.7 million [1]. English walnut trees in California are typically grown on interspecific hybrid rootstocks that promote vigor and productivity. Crown gall (caused by *Agrobacterium tumefaciens*), *Phytophthora* crown and root rot (*Phytophthora* spp.) and nematode root lesions (*Pratylenchus vulnus*) are important rootstock diseases of California walnuts. *Agrobacterium tumefaciens* is a rod-shaped, Gram-negative soil bacterium, while *Phytophthora* spp. is a soilborne *Oomycete*. These microbes obstruct the vascular tissues with tumors (*A. tumefaciens*) or kill root tissues directly (*Phytophthora* spp.). Both pathogens cause disease by inhibiting the flow of nutrients and water to the scion (upper portion of the plant), reducing tree productivity while imperiling its health. *Pratylenchus vulnus*, an obligate endoparasitic migratory nematode, causes yield loss by damaging the root cortical tissue and consuming plant nutrients, which ultimately impedes the flow of nutrients and water to the scion.

Walnut growers spend substantial resources managing these phytopathogens. According to the 2017 UC Davis walnut production cost study, management costs for nematode suppression were USD 1400 per acre before planting and USD 97 per acre per year for *Phytophthora* [2]. Costs to manage *A. tumefaciens* need to be more comprehensively estimated but include labor for the surgical removal of tumors and follow-up topical treatments to prevent redevelopment.

Despite current agronomical practices and treatments, these phytopathogens continue to cause severe yield losses. In one study, *A. tumefaciens* infection led to crop losses of 25% to 50% per infected tree or the entire loss of the infected tree [3]. Another study found crown gall disease caused a 12% decrease in cumulative four-year yield per 25% of the galled trunk diameter [4]. Aggressive species of *Phytophthora*, such as *P. cinnamomi*, can decimate most trees in walnut orchards infested with the pathogen unless a resistant rootstock is used [5,6]. To date, ‘RX1′ is the only commercially available rootstock that exhibits sufficient resistance to this pathogen [7]. Yield and/or survivability data for *P. vulnus* are less detailed but estimated at 15–20% and, in severe cases, the total failure of an orchard. Phytothagous nematodes were estimated to be present in 85% of walnut orchards [8]. It has been estimated that root system diseases cost the California walnut industry USD 241 million per year, an industry with an annual production value of approximately USD 1.24 billion [9].

Another trait of interest is tree vigor. From the grower’s perspective, if trees become too large, they are hard to manage. For example, plant-protective sprays against pests and diseases become less effective as the tree grows larger due to the physical limitations of the equipment’s performance. Moreover, harvest can be more difficult as the trees grow larger. Conversely, low vigor can be problematic in that the tree needs to reach a minimum size to bear fruit that is worth harvesting. Even in a dwarf orchard scenario, the trees must be planted closer together to maximize the efficiency of available sunlight usage compared to an orchard with higher vigor trees. It is also suspected that so-called ‘high-density’ plantings would be vulnerable to increased pest and disease pressure.

English walnut scions are grafted to rootstocks to physically combine benefits of the scion genotype, e.g., high nut quality, with beneficial traits of the rootstock genotypes. Early in California walnut production, selections of Northern California black walnut (*Juglans hindsii*) or English walnut (*J. regia*) rootstocks highly susceptible to *Phytophthora* spp. and *P. vulnus* were used. In the early 1900s, Luther Burbank created the first hybrid walnut rootstock seedlings by crossing *J. hindsii* with *J. regia* [10]. Burbank termed the hybrid ‘paradox’ due to its unusual vigor and other ‘anomalies.’ Paradox became the most popular walnut rootstock due to its vigor and greater, though still limited, resistance to *Phytophthora* spp. and *P. vulnus*, compared to *J. hindsii* and *J. regia* [10]. Paradox is now understood to include any cross of a black walnut species (*J*. sect. *Rhysocaryon*) × *J. regia*. The currently most popular California scion cultivar, *J. regia* cv. ‘Chandler’ is highly susceptible to *A. tumefaciens*, *Phytophthora* spp. and *P. vulnus*; thus, the bare root planting of ‘Chandler’ is almost never considered. Given that the nut quality of Paradox is considered unacceptable, grafting is the most popular choice. Growers have looked to genetic resistance of rootstocks as a foundation for the management of soilborne disease challenges. Interdisciplinary walnut rootstock breeding efforts led to the discovery of ‘RX1′ and ‘VX211′ rootstocks. Clonal Paradox ‘RX1′ (*Juglans microcarpa* × *J. regia*) is now the rootstock of choice when *Phytophthora* and *A. tumefaciens* are of concern due to its higher resistance to these pathogens [7,11]. Another clonal Paradox, ‘VX211′ (*J. hindsii* × *J. regia*), is the preferred rootstock when *P. vulnus* is a problem due to its greater tolerance than other commercially available rootstocks [12]. In current breeding work, selections from Paradox-type crosses are made with the goal of creating elite hybrids triple resistant to *A. tumefaciens*, *Phytophthora* spp. and *P. vulnus* infection. When analyzing breeding populations of *Juglans microcarpa* × *J. regia*, hybrids were discovered with improved resistance to *A. tumefaciens* and *Phytophthora* spp. [7,13].

In addition to traditional breeding, molecular approaches to walnut improvement are progressing. RNA interference designed to target key genes of *A. tumefaciens* and *P. vulnus* in walnuts resulted in resistance to these pathogens in vitro [14,15,16]. The publication of high-quality genomes of *J. microcarpa* and *J. regia* facilitated genomic and functional genomic analyses of the *J. microcarpa* × *J. regia* hybrids mentioned previously [17,18]. *Juglans microcarpa* is a short-statured black walnut native to riparian areas of North America and its genome size and chromosome number are very similar to *J. regia* [18]. QTL analysis of these hybrids showed a significant region on chromosome 4D of *J. microcarpa* correlated with *Phytophthora* spp. and *A. tumefaciens* resistance [19]. These QTL results provided the first insights into the genetic basis of resistance to these key pathogens in walnut. These studies accelerated walnut rootstock development efforts for the industry. QTL analysis can provide valuable information on what genomic loci may be involved in phenotypes but gives little information on the genes involved or on their expression levels.

The work that we report here complements the previous efforts through the use of a functional genomics approach. We hypothesized that genes associated with phenotypes of susceptibility, resistance or vigor could be identified using RNA-seq of unchallenged plant tissue. Very little is understood about the molecular biology of basal host resistance or vigor in walnut rootstocks. The objectives of this research were to conduct an RNA-seq experiment (i) to determine loci with expression profiles related to basal resistance to *A tumefaciens*, *Phytophthora* spp. and *P*. *vulnus*; and (ii) to estimate the physiological function of these loci.

## 2. Results

### 2.1. Differential Expression Analysis

The roots of seven unchallenged *J. microcarpa* × *J. regia* hybrids were selected to represent a range of phenotypes for vegetative vigor and resistance to *A. tumefaciens*, *Phytophthora* spp. and *P. vulnus*. The phenotype units for “vigor” were expressed tree-height at three years; whereas the units for the response phenotype to *A. tumefaciens* and *Phytophthora* spp. were expressed as disease severity scores (with ranges of 1 to 4 and from 0 to 100, respectively) and the units of the response phenotype to *P. vulnus* were expressed as counts of nematode per gram of root. To identify putative transcripts associated with these phenotypes, we performed RNA-seq analysis on seven unchallenged *J. microcarpa* × *J. regia* hybrid progeny.

**Figure 1:**
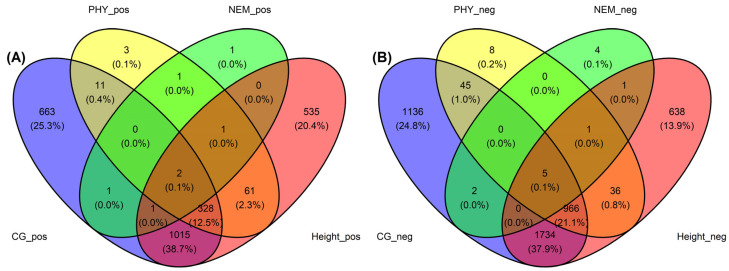
(**A**) Venn diagram of differentially expressed genes positively correlated with the *A. tumefaciens* (CG, blue), *Phytophthora* spp. (PHY, yellow), *P. vulnus* (NEM, green) and tree height at three years (height, pink) phenotype scores. (**B**) Venn diagram of differentially expressed genes negatively correlated with the *A. tumefaciens* (CG), *Phytophthora* spp. (PHY), *P. vulnus* (NEM) and tree height at three years (height) phenotype scores.

**Figure 2:**
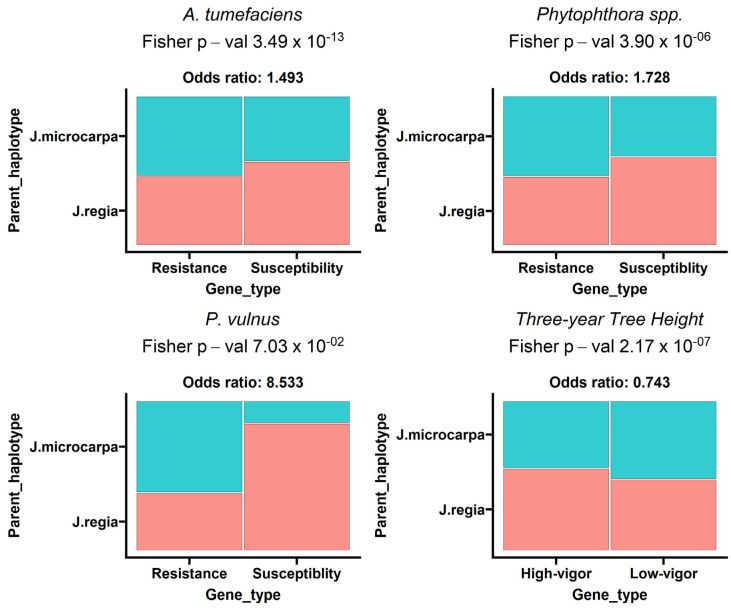
Mosaic plot representing proportions of differentially expressed genes (DEGs) from each trait colored by the haplotype the genes mapped to. Each plot is labeled with the pathogen, Fisher’s exact *p*-value and Fisher’s exact odds ratio. The odds ratio represents the ratio of the odds of the *J. regia* haplotype expressing a gene positively correlated with the trait compared to the odds of the *J. microcarpa* haplotype expressing a gene negatively correlated with the trait.

Principal component analysis (PCA) of the counts per million (CPM) normalized reads revealed that PC2 strongly correlated with *A. tumefaciens* phenotypic response (Table 1). Tree vigor showed a strong trending correlation with PC1 but was not significant (*p*-value 5.69 × 10^−2^). Modeling of the expressed genes against each trait revealed hundreds of differentially expressed genes (DEGs), with many DEGs shared across traits (Figure 1). We observed 3888 genes negatively associated, 2021 genes positively associated and 13,290 genes not associated with the *A. tumefaciens* phenotypic response; 1061 genes negatively associated, 407 genes positively associated and 17,731 genes not associated with the *Phytophthora* spp. phenotypic response; 13 genes negatively associated, 7 genes positively associated and 17,962 genes not associated with the *P. vulnus* phenotypic response; and 3381 genes negatively associated, 1943 genes positively associated and 12,658 genes not associated with tree height (Table 2). Modeling of the *P. vulnus* phenotypic response yielded substantially fewer DEGs than the other traits (Table 2). In each trait analyzed, the odds of expressing genes positively associated with the trait were slightly higher in the *J. regia* haplotype compared to the *J. microcarpa* haplotype. Conversely, the odds of expressing genes negatively associated with the phenotype were slightly higher in the *J. microcarpa* haplotype compared to that of the *J. regia* haplotype. (Figure 2). Regardless of haplotype, the number of differentially expressed genes negatively associated with each trait was always greater than the number of differentially expressed genes positively associated with each trait (Table 2). The full results of the differential expression analysis are available in Supplementary File S3.

### 2.2. Biological Process Analysis

**Figure 3:**
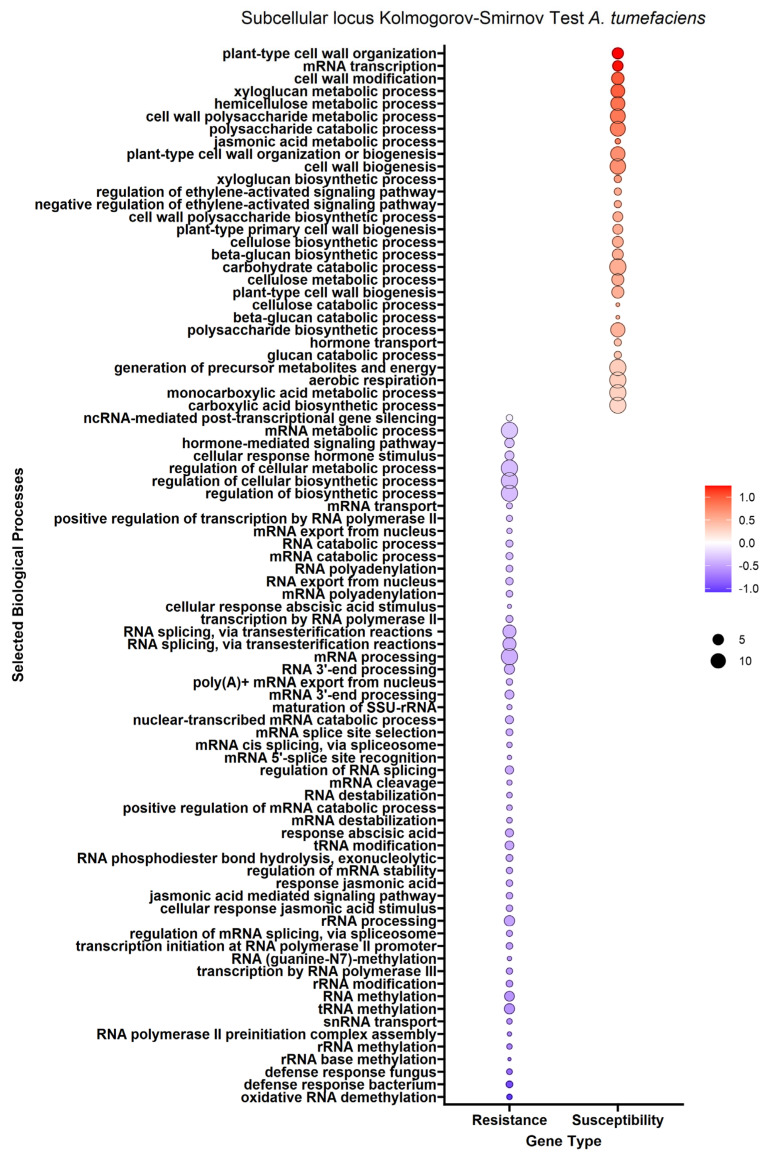
Enrichment analysis of gene ontology terms mapped to differentially expressed genes (DEGs) for the *A. tumefaciens* phenotypic response. Each point represents a biological process term. Point color represents the average log fold change in expression per biological process; point size represents significance in log10(fdr) × −1. See Section 4 for term labeling.

**Figure 4:**
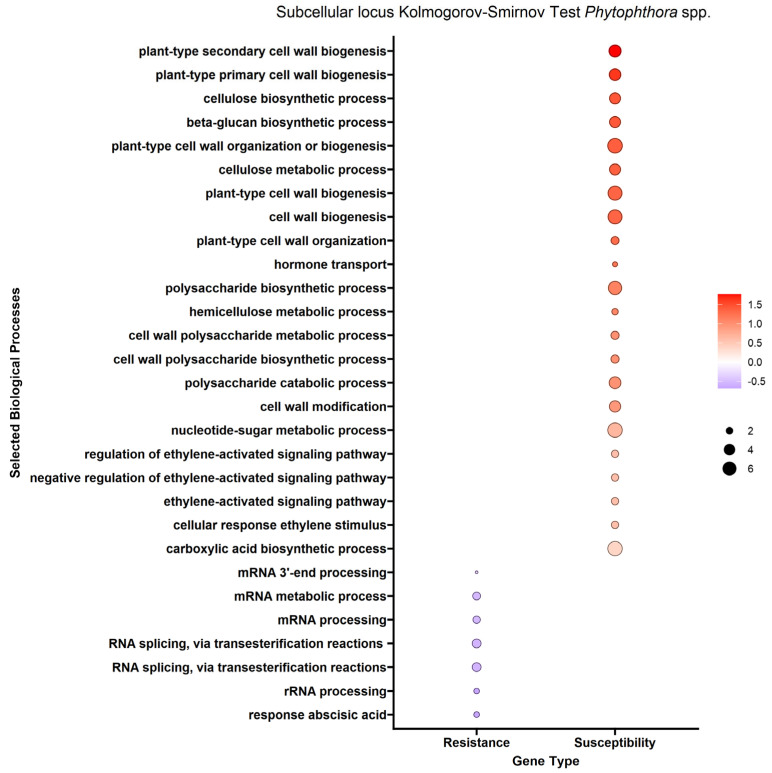
Enrichment analysis of gene ontology terms mapped to differentially expressed genes (DEGs) for the *Phytophthora* spp. phenotypic response. Each point represents a biological process term. Point color represents the average log fold change in expression per biological process; point size represents significance in log10(fdr) × −1. See Section 4 for term labeling.

**Figure 5:**
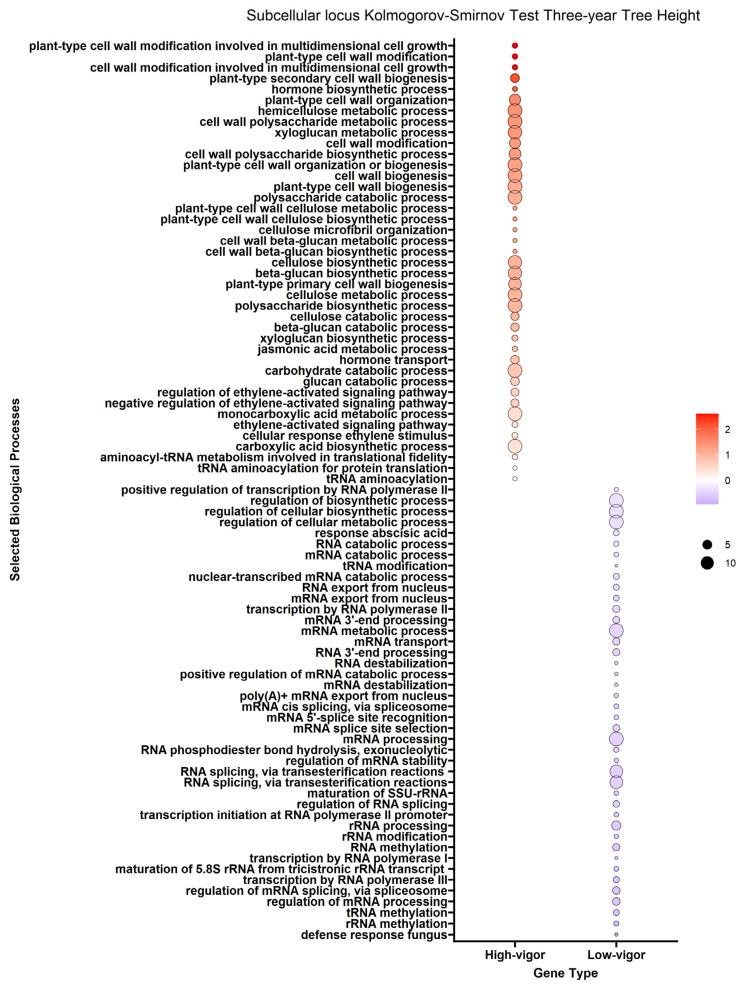
Enrichment analysis of gene ontology terms mapped to differentially expressed genes (DEGs) for tree height. Each point represents a biological process term. Point color represents the average log fold change in expression per biological process; point size represents significance in log10(fdr) × −1. See Section 4 for term labeling.

We performed enrichment analysis using Kolmogorov–Smirnov tests on the DEGs to reveal gene ontology biological processes (BPs) enriched in the DEGs of each trait. The enrichment analysis resulted in 2958 genes mapped to 322 biological processes for the *A. tumefaciens* phenotypic response, 464 genes mapped to 114 biological processes for the *Phytophthora* spp. phenotypic response, no significant results for *P. vulnus* and 2605 genes mapped to 296 biological processes for tree height (Figure 3, Figure 4 and Figure 5). The differences in the number of enriched terms likely reflected the number of genes in the input of the analysis. Transcripts positively correlated with each trait were enriched in BPs involved in cell wall organization, polysaccharides, glucans and cellulose. Genes that mapped to these terms included cellulose synthases, fasciclin-like arabinogalactan proteins and xyloglucan endotransglucosylase/hydrolases (File S1). Conversely, transcripts negatively correlated with each trait were enriched in some form of RNA metabolic process. The regulation of RNA metabolic processes is defined as “Any process that modulates the frequency, rate or extent of RNA biosynthetic process (http://amigo.geneontology.org/amigo/term/GO:0051252 (accessed on 6 June 2023)”. “Defense response to bacterium” was significantly enriched with transcripts negatively correlated with the *A. tumefaciens* phenotypic response (Figure 3). No terms mentioning defense response were significantly enriched in the *Phytophthora* spp. phenotypic response, *P. vulnus* phenotypic response, or tree height. “Jasmonic acid-mediated signaling pathway”, “cellular response to jasmonic acid stimulus”, and “response to jasmonic acid” were enriched with transcripts negatively correlated with the *A. tumefaciens* phenotypic response (Figure 3). These terms were not enriched in the other traits. Moreover, several terms mentioning ethylene were enriched with transcripts positively correlated with the *A. tumefaciens* phenotypic response, *Phytophthora* spp. phenotypic response and tree height (Figure 3, Figure 4 and Figure 5). “Response to abscisic acid” was enriched with transcripts negatively correlated with the *A. tumefaciens* phenotypic response and tree height (Figure 3 and Figure 5). The expression of genes associated with the crucial plant hormone term salicylic acid was not enriched in any analysis (File S1). However, “hormone-mediated signaling pathway” and “cellular response to hormone stimulus were enriched with transcripts negatively correlated with the *A. tumefaciens* phenotypic response and “hormone transport” was enriched with transcripts positively correlated with tree height (Figure 3 and Figure 5).

### 2.3. Subcellular Localization Analysis

**Figure 6:**
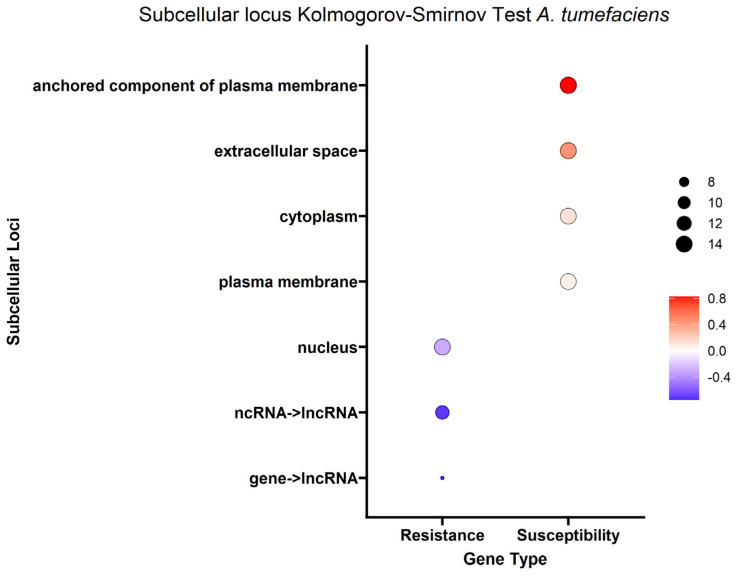
Enrichment analysis of BUSCA subcellular loci mapped to differentially expressed genes (DEGs) for the *A. tumefaciens* phenotypic response. Point color represents the average log fold change in expression per biological process; point size represents significance in log10(fdr) × −1.

**Figure 7:**
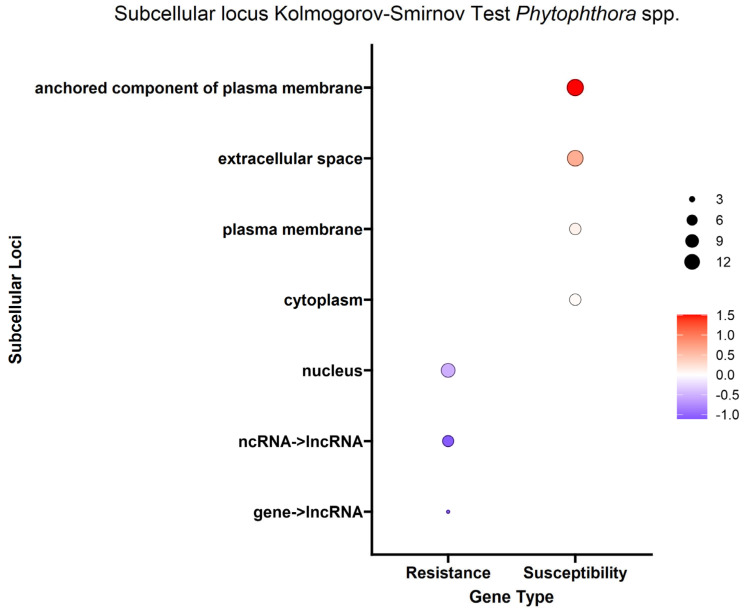
Enrichment analysis of BUSCA subcellular loci mapped to differentially expressed genes (DEGs) for the *Phytophthora* spp. phenotypic response. Point color represents the average log fold change in expression per biological process; point size represents significance in log10(fdr) × −1.

**Figure 8:**
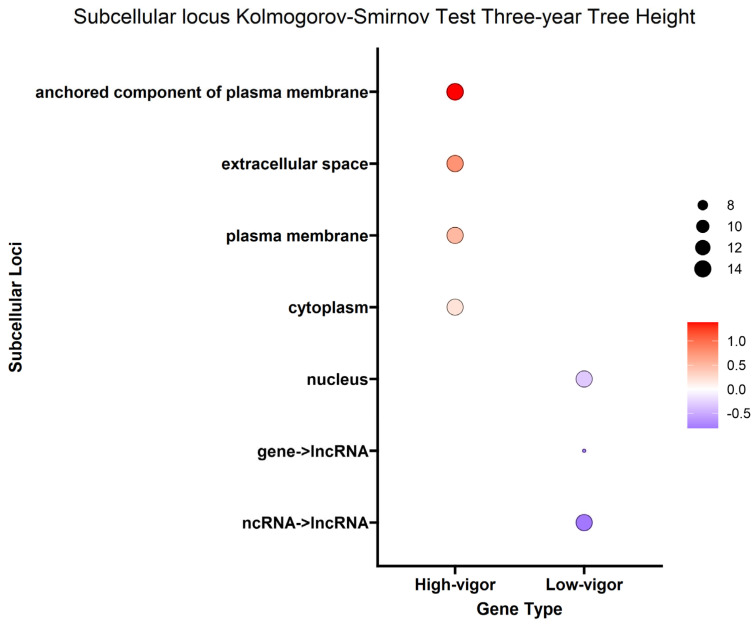
Enrichment analysis of BUSCA subcellular loci mapped to differentially expressed genes (DEGs) for tree height. Point color represents the average log fold change in expression per biological process; point size represents significance in log10(fdr) × −1.2.4. Splicing Analysis.

Given that genes encode proteins and proteins often have subsequences that determine where they will go in or outside the cell (subcellular localization), we were able to estimate where the protein product of each DEG would go in the cell. To better understand the overall localization of the DEG protein products within the cell, the computationally predicted subcellular localization of each set of DEG protein products was analyzed. As with the biological process analysis, enrichment analysis using the Kolmogorov–Smirnov test was conducted on the subcellular localizations for the DEG protein products. Using a false discovery rate (FDR) threshold of 0.05 for all traits, the plasma membrane, anchored component of plasma membrane and extracellular space were enriched in DEG protein products positively correlated with the *A. tumefaciens*, *Phytophthora* spp. and vigor phenotypes (Figure 6, Figure 7 and Figure 8). The cytoplasm was enriched only in the DEG protein products positively correlated with *A. tumefaciens* and the plasma membrane was enriched only in DEG protein products positively correlated with the *Phytophthora* spp. and vigor phenotypes (Figure 6, Figure 7 and Figure 8). We observed no significant enrichment in subcellular loci for the *P. vulnus* phenotypic response. Alternatively, a trend of targeting the nucleus was observed for DEG protein products negatively correlated with each trait except for the *P. vulnus* phenotypic response (Figure 6, Figure 7 and Figure 8). We included long non-coding RNAs in this analysis and this class was enriched with transcripts negatively correlated with each trait except for the *P. vulnus* phenotypic response. The full results of this analysis can be found in File S2.

**Figure 9:**
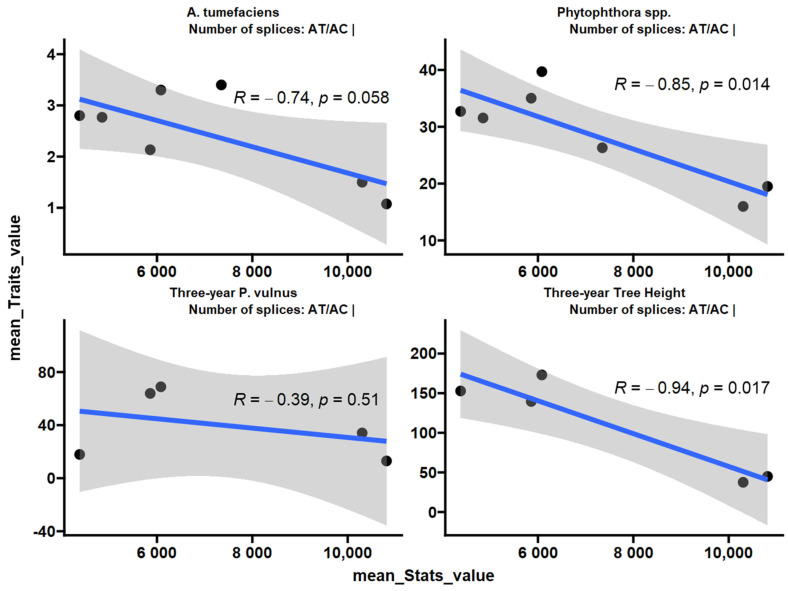
Pearson correlation of number of AT/AC splices with each trait. Splicing data were extracted from the “log.final.out” file from the STAR aligner output. The blue line is a trend line fitted to the data using a linear model and the grey shading is the standard error.

Given that increased expression of splicing-annotated transcripts was associated with disease resistance and low vigor, we analyzed the number of splices determined by the STAR aligner. The number of AT/AC splices had a negative correlation with the *Phytophthora* spp. phenotypic response and vigor (Figure 9). A similar trend was observed for the *A. tumefaciens* phenotypic response with a *p*-value of 0.058. AT/AC splices were not correlated with the *P. vulnus* phenotypic response. The number of GC/AG, GT/AG, total, non-canonical or annotated (sjdb) splices did not correlate with any trait in this study.

### 2.4. QTL Region Analysis

QTLs for *A. tumefaciens* and *Phytophthora* spp. have been reported to peak between markers 31.01_Jm4D_26359154 and 31.01_Jm4D_26669075 for the breeding population our samples were derived from [19]. We analyzed the DEGs within this region of the *J. microcarpa* genome and found three transcripts negatively correlated with *A. tumefaciens* phenotypic response and three transcripts negatively correlated with vigor. No DEGs from the *Phytophthora* spp. or *P. vulnus* phenotypic responses were found in this region. Both small nuclear ribonucleoprotein SmD3b (NCBI ID 121260033) and the pre-rRNA-processing protein TSR1 homolog (NCBI ID 121259960) were annotations for transcripts negatively correlated with *A. tumefaciens* and vigor traits in this region of chromosome 4D (Table 3). Probable acyl-activating enzyme 1, peroxisomal (NCBI ID 121259974), was an annotation for a transcript negatively correlated uniquely with vigor, and dolichol-phosphate mannosyltransferase subunit 1 (NCBI ID 121260019) was an annotation for a transcript negatively correlated uniquely with *A. tumefaciens* (Table 3).

## 3. Discussion

This study follows up on a previous QTL analysis, which showed that resistance to *A. tumefaciens*, *Phytophthora cinnammomi* and *Phytophthora pini* mapped to a ~300 kb region on chromosome 4D of the *J. microcarpa* genome [19]. With a small subset of the hybrids from the QTL study, we regressed the expressed genes against *A. tumefaciens*, *Phytophthora* spp., *P. vulnus* and tree height (vigor) phenotypes. The summary of our results suggests cell wall biogenesis is involved in vigor and pathogenesis caused by *A. tumefaciens* and *Phytophthora* spp. More specifically, increased cell wall biogenesis results in susceptibility to these pathogens and increased vigor, while decreased cell wall biogenesis results in resistance and decreased vigor. Moreover, we found several DEGs within the ~300 kb region of chromosome 4D that may be causal for these phenotypes. Lastly, it is important to compare the similarity of this study to [19]. While this study is not considered to be a GWAS due to the sample size, we also observed co-location of quantitative trait biological processes.

### 3.1. Uninfected Transcriptional Repertoires May Predict Susceptibility

One component from the PCA of the gene counts correlated strongly with *A. tumefaciens* gall size in this study (Table 1), suggesting that the group of transcripts was critical for the host response. The correlation between tree height and PC1 was strong but, with two fewer replicates than *A. tumefaciens* or *Phytophthora* spp., it did not meet the significance threshold (Table 1, Figure S1). Given this result, RNA-seq may be able to predict disease outcomes. The use of RNA-seq for the genomic prediction of traits in plants has been successful for carotenoid and seed oil biosynthesis in sweet corn and rapeseed, respectively [20,21]. This result suggested that there is potential for deciphering the mechanisms underlying phenotypic phenomena and using the expression of candidate genes as biomarkers for genomic selection.

### 3.2. Low Differential Expression Signal in P. vulnus

While all other traits analyzed in this study showed a strong signal in the expression data, modeling against *P. vulnus* only resulted in a handful of DEGs. The top resistance DEG had an annotation of E3 ubiquitin-protein ligase UPL1-like and the top susceptibility DEG had an annotation of endoplasmic reticulum–Golgi intermediate compartment protein 3-like. These genes and others should be further analyzed for fitness as candidate genes to be altered in genetic experiments to confirm their association with *P. vulnus* susceptibility. There could be many reasons for the low signal. Perhaps the *P. vulnus* phenotype is mediated only by a few genes. Another possibility is that resistance to nematodes is induced upon nematode attack, which we cannot assess, given the experimental design of this study. Also, *P. vulnus* susceptibility was phenotyped by counting the number of nematodes per gram of root and, thus, was not a direct measure of the host response. That is, the root lesions caused by *P. vulnus* were not measured, but the nematode population was. Also, nematode numbers are notoriously variable and thus offer relatively low precision. The phenotyping for all other traits in this study involved direct measures of the host, such as the percent crown and root rot due to *Phytophthora* spp., the size of the gall caused by *A. tumefaciens*, or tree height. Given that we analyzed biochemicals (mRNAs) of the host, it is sensible that direct measures of the host phenotypes would garner a greater signal in mRNAs than nematode counts, which may not correlate well with the root lesions caused by *P. vulnus*.

### 3.3. J. regia May Be a Source of Vigor and Susceptibility

The use of a combined genome reference in our differential expression analysis revealed the expression of significantly more transcripts positively correlated with each susceptibility and vigor trait from the *J. regia* haplotype than from the *J. microcarpa* haplotype (Figure 2). The *P. vulnus* phenotypic response was not significant in this analysis. Conversely, the *J. microcarpa* haplotype expressed significantly more transcripts negatively correlated with each trait. This seems consistent with the fact that, initially, own-rooted plantings of English walnuts (*J. regia*) were soon found to be susceptible to *A. tumefaciens*, *Phytophthora* spp. and *P. vulnus*. To remedy this problem, rootstocks sourced from *J. hindsii* were used [10]. Eventually, hybrids of *J. hindsii* × *J. regia* and *J. microcarpa* × *J. regia* were introduced for improved disease tolerance and, in the former, at least, vigor. Therefore, *J. regia* seems to be a source of disease susceptibility in rootstocks, which is consistent with our RNA-seq analysis. On a different note, significantly more genes associated with high vigor were expressed from the *J. regia* haplotype than from the *J. microcarpa* haplotype (Figure 2), indicating that it may be contributing more to the height of the hybrids than *J. microcarpa*. High-vigor trees seem to be preferred in the California walnut industry, or at least a minimum amount of vigor is required for realistic productivity. Perhaps there is a “middle ground” between the contribution of each haplotype to gene expression as it relates to agronomic desirability.

### 3.4. Defense Response and Hormones Involved

One potential pathogen resistance mechanism could be the upregulation of genes associated with defense responses, such as the family of pathogenesis-related (PR) genes, nonexpresser of PR genes (NPR) genes, or any plant hormone responses. Genoontology.org defines “defense response” as “Reactions, triggered in response to the presence of a foreign body or the occurrence of an injury, which result in restriction of damage to the organism attacked or prevention/recovery from the infection caused by the attack”. A complete list of genes associated with this term when filtered for “*Juglans regia*” can be found at http://amigo.geneontology.org/amigo/term/GO:0006952 (accessed on 8 July 2023). The walnut expression data suggested that the resistance mechanism may involve defense-related genes or plant hormones. We observed that “Defense response to bacterium” was significantly associated with resistance and not susceptibility in *A. tumefaciens* (Figure 3). This result is unsurprising given that *A. tumefaciens* is a bacterium. Oddly, no terms mentioning the defense response were significantly enriched in *Phytophthora* spp. or *P. vulnus*. Interestingly, jasmonate signaling was associated exclusively with resistance to *A. tumefaciens* (Figure 3). A recent review on jasmonic acid in plants reports protective effects of both endogenous and exogenous jasmonic acid against necrotrophic pathogens [22]. Therefore, it is possible that jasmonic acid signaling is contributing to resistance to *A. tumefaciens* in these hybrids. Moreover, several terms mentioning ethylene were enriched with transcripts positively associated with *A. tumefaciens* and *Phytophthora* spp. phenotypic responses and vigor, suggesting roles for ethylene in susceptibility to these pathogens and increased vigor (Figure 3, Figure 4 and Figure 5). The literature of ethylene’s effect on plant growth seems mixed as plants can respond with increased or decreased growth depending on the species and concentrations analyzed [23]. Similar trends have been noted for ethylene’s role in defense, where treatment with ethylene or its inhibitor, or mutants with impaired ethylene signaling, elicited conflicting results depending on the plant and pathogen combinations [24,25]. Our results also suggested abscisic acid is involved in resistance to *A. tumefaciens* and decreased vigor (Figure 3). Like ethylene, abscisic acid’s effects on plant–pathogen interactions are mixed [26]. Of course, abscisic acid’s role in plant growth is clear; for example, it regulates stomatal closure and thus limits growth at higher concentrations by limiting carbon acquisition [27]. This matches well with the observations that abscisic acid-related genes were highly expressed in low-vigor hybrids, potentially resulting in greater stomatal closure and less growth (Figure 5).

### 3.5. DEGs within the QTL Region. Is RNA Splicing Involved?

We found four unique DEGs associated with resistance to *A. tumefaciens* and decreasing tree height within the QTL region reported in [19]. The expression of two of these genes, small nuclear ribonucleoprotein SmD3b and pre-rRNA-processing protein TSR1 homolog, were shared with resistance to *A. tumefaciens* and decreasing tree height (Table 3). The small nuclear ribonucleoprotein SmD3b is involved in RNA splicing and the pre-rRNA-processing protein TSR1 homolog is involved in ribosome biogenesis [28,29]. This result aligns with the fact that RNA processing and splicing-annotated transcripts were associated with resistance. Unsurprisingly, we also see that AT/AC splicing events correlated with resistance. Given that these genes are within the QTL region and are differentially expressed, they are likely candidates for the gene(s) causal for resistance to *A. tumefaciens* and modulators of tree vigor. Moreover, if these are genes with SNPs associated with resistance or are being affected by said SNPs, perhaps they are causing the global change in RNA splicing and processing that is associated with resistance as they are genes involved in such processes. We further hypothesize that RNA splicing may affect cell wall biogenesis, as we observed that the two processes are inversely related.

### 3.6. Potential Link between Cell Wall Biogenesis, Pathogenesis and Vigor

Cell wall biogenesis-annotated transcripts were linked to increased disease susceptibility and plant vigor in the linear modeling and enrichment analysis of the DEGs. These results are consistent with the relevant literature on cell walls. Cell wall composition, determined in part by the expression of cell wall enzymes, is fundamental to a plant’s physical characteristics, such as disease resistance or vigor [30]. Plant cell walls are perhaps the most critical component of plant immunity and always the first obstacle a pathogen must overcome. Host manipulation, carbon acquisition, effector delivery and defense response suppression often require breaching the cell wall [31]. Perhaps the susceptible hybrids in our study have weaker cell walls, somehow related to the increased expression of cell wall-annotated transcripts. Cellulose, hemicelluloses (like xyloglucan and xylan), pectins and cell wall proteins, in order of abundance, represent the predominant constituents of the cell wall [32,33]. We observed several DEGs with annotations directly involved in these constituents’ biosynthesis, whose expression correlates with susceptibility and high vigor (File S1). Highlighting the importance of these polysaccharides are the myriad of cell wall degrading enzymes that many pathogens use to breach this barrier. These enzymes are key virulence factors and include enzymes such as cellulase, xylanase, pectin methylesterase, polygalacturonase and pectate lyase [34].

In terms of vigor, every plant cell in a growing tissue must expand in volume to achieve this growth. For example, meristematic xylem cells can expand >30,000 times their original size and superficial cotton hair cells elongate to 1000 times their original size before maturity [35]. Plant cell walls determine the functional properties, shape, communication and overall expansion and growth of plant cells [36]. The cell wall must undergo biogenesis in order to cope with such expansion. Thus, it is sensible that we observe the increased expression of transcripts annotated with cell wall biogenesis as plant growth increases.

Our subcellular localization results further support the hypothesis that cell wall biogenesis is involved in the pathogenesis of *A. tumefaciens* and *Phytophthora* spp. and vigor in walnuts. The DEG protein products positively associated with *A. tumefaciens* and *Phytophthora* spp. phenotypic responses and vigor targeted significantly more cellular barriers, such as the plasma membrane, its anchored components and the extracellular space, than the DEG protein products negatively associated with *A. tumefaciens* and *Phytophthora* spp. phenotypic responses and vigor (Figure 6, Figure 7 and Figure 8). The extracellular space includes the cell wall, and the plasma membrane is directly adjacent to the cell wall. In other words, significant increases in the expression of DEG protein products targeting the cell wall occurred as the plants became taller and more susceptible to these pathogens. In the biological processes and subcellular loci associated with the up genes, the increased expression of genes associated with cell wall biogenesis (e.g., cellulose synthase, xyloglucan endotransglucosylase, arabinogalactan proteins) resulted in increased vigor and susceptibility to *A. tumefaciens* and *Phytophthora* spp. (Figure 3, Figure 4 and Figure 5, Supplementary Files S1 and S2). The predicted functions, biological processes and subcellular localizations of these up genes strongly suggest that the increased activity of cell wall biosynthetic enzymes contributes to vigor and pathogen susceptibility in these hybrids.

### 3.7. Cell Wall Biosynthetic Genes Are Known to Affect Pathogenesis and Plant Growth

Tree height, *A. tumefaciens* phenotypic response and *Phytophthora* spp. phenotypic response are strongly correlated in our phenotypic data (Table 4). Therefore, it is not surprising that the results for each trait are similar. In other words, tree height, *A. tumefaciens* phenotypic response and *Phytophthora* spp. phenotypic response may be linked through a common mechanism relating to the expression of cell wall biogenetic genes. Alterations in cell wall biogenesis can affect a plant’s susceptibility to pathogens. For example, in *Arabidopsis thaliana*, mutations in five genes necessary for cellulose synthesis in primary cell walls resulted in enhanced resistance to *Fusarium oxysporum*, a root-infecting hemibiotrophic fungal pathogen [37]. These genes included *cesa3-3*, *cobra-6*, *ctl1-2*, kor*1-4* and *prc1-1*. *Cesa3-3 and prc1-1* encode cellulose synthases [38,39]. *Ctl1-2* encodes an apoplastic chitinase-like protein regulating cellulose biosynthesis [40]. *Cobra-6* affects cellulose biosynthesis; its mechanism is nebulous but it may interact with the orientation and synthesis of cellulose microfibrils [41]. *Kor1-4* encodes a plasma membrane-bound endo-1,4, -β-glucanase [42]. A significant phenotype of these mutants is cellulose deficiency, which is likely involved in enhanced resistance to *F. oxysporum*. While we did not measure cellulose accumulation in the hybrids studied here, the transcriptomic data indicated positive relationships between the expression of cellulose biosynthetic enzymes and susceptibility to all diseases (Figure 3, Figure 4 and Figure 5, File S1). Moreover, the transcriptional changes in the *Fusarium*-resistant *A. thaliana* mutants mirrored that of our resistant hybrids. Specifically, we observed reduced expression of many *cesas* (cellulose synthases) and *cobras* (protein COBRA) in the resistant walnut hybrids (Files S1). The downregulation of cell wall biosynthetic transcripts was observed in RNAs of wild-type *A. thaliana* challenged with *F. oxysporum* vs. unchallenged [37]. Interestingly, the impairment of cellulose synthases in *A. thaliana* also impairs growth [37,43]. In fact, mutations in *cobra-6*, *ctl1-2* and *kor1-4* all resulted in reduced plant growth [40,41,42]. Our observations of transcripts associated with vigor agree very well with this, suggesting that increasing expression of cell wall biosynthetic genes results in increasing vigor in walnuts (File S1). Moreover, we also hypothesize a potential link between walnut vigor and disease resistance. This could be tested by knocking out a cell wall biosynthetic gene or inhibiting its protein product, hypothetically resulting in reduced growth and increased disease resistance.

While it is functional to understand how reduced cellulose biosynthesis affects plant growth, the mechanism of its role in disease resistance is less clear. Perhaps the recomposition of cell wall polysaccharides creates a matrix that is more resistant to pathogenic cell wall degrading enzymes. For example, the proportion of pectin or hemicellulose could increase relative to that of cellulose, changing the characteristics of the cell wall considerably and possibly making a pathogenic cellulase less effective. Moreover, the degree of branched polysaccharide cross-linking could impact these cell wall characteristics by affecting their architectural stability.

The arabinogalactan proteins are another gene family significant for cell wall biogenesis, growth and potentially pathogen defense [31]. In *Arabidopsis*, downregulation of the arabinogalactan protein gene *AtAGP17* resulted in a decreased efficiency of *Agrobacterium* transformation [44]. In microscopic investigation of the *Arabidopsis AtAGP17* mutant, *A. tumefaciens* could not bind to its root surface compared to wild-type *A. thaliana* roots. Moreover, nonspecific inhibition of arabinogalactan proteins with the Yariv reagent also reduced *A. tumefaciens* transformation of *Arabidopsis* roots [44]. Like the other cell wall biogenesis-related genes mentioned above, arabinogalactan protein knockouts tend to result in reduced growth or growth defect phenotype and the growth of plants on Yariv-reagent-containing media is also reduced [44,45]. Similarly, in our analysis, two transcripts encoding arabinogalactan proteins were underexpressed in resistant and low-vigor hybrids (File S1). Perhaps the expression of these genes is contributing to our observed phenotypes.

## 4. Methods and Materials

### 4.1. Plant Material

Unchallenged, previously propagated and evaluated *J. microcarpa* × *J. regia* hybrid clonal genotypes varying in vigor (measured as 3-year field height) and resistance to *A. tumefaciens*, *Phytophthora* spp. and *P. vulnus*, were chosen for use in this experiment. Additional plants of the clonal genotypes MS1-36, MS1-41, MS1-56, MS1-122, STJM-4, 29JM-11 and JMS-12 were developed from tissue culture and grown in pots in a greenhouse for six months. These progenies were selected from two breeding populations of seedling genotypes. One population of 368 individuals was derived from a cross between the *J. microcarpa* mother tree 31.01 and *J. regia* father tree “Serr”. The other population of 42 individuals was derived from a cross between the *J. microcarpa* mother tree 29.11 and *J. regia* father tree “Serr”. The hybrids MS1-36, MS1-41, MS1-56 and MS1-122 are progeny from the 31.01 × “Serr” cross, and 29JM-11, JMS-12 and STJM-4 are progeny from the 29.11 × “Serr” cross. The details of how these hybrids were produced have been described in detail [19].

### 4.2. Phenotypic Analysis

The phenotyping of these hybrids for resistance to *A. tumefaciens* and *Phytophthora* spp. has been described in detail [19]. Briefly, the pathogen-resistance phenotype used for *A. tumefaciens* was a score on a scale of one (no gall) to four (complete girdling of the plant from gall). The pathogen-resistance phenotype used for *Phytophthora* spp. was a visually assessed percentage of crown or root rotted after pathogen inoculation of either *P. cinnamomi* or *P. pini*. The average percentages of crown and root rot for *P. cinnamomi* and *P. pini* were entered into the analysis and were then referred to as “*Phytophthora* spp.”.

To phenotype hybrids for *P. vulnus* resistance or susceptibility, at least eight clonal saplings of each hybrid and commercial comparatives RX1 and VX211 [11,46] were planted in field plots in March 2014. The hybrids to be used in this study were propagated from tissue cultures and developed into saplings in greenhouse culture. At the Kearney Agricultural Research and Extension Center (36.6015° N, 119.5109° W), saplings were planted in their genotype group in rows of 3.35 m distance at 1.65 m spacing within the row. About one month after planting, every tree was concomitantly inoculated with ~1000 vermiform *P. vulnus* and second-stage juveniles (J2) of *Meloidogyne incognita* by dispensing infested field soil from underneath infected perennial crops at the base of the tree. Selected trees of the genotype groups were chosen for root collections for nematode evaluations. A 20–25 cm-deep trench was dug next to the tree to collect young roots of the respective tree genotype avoiding suberized roots. Kept cool in plastic bags until processing within 48 h of collection, the roots were chopped into 1.2 cm pieces and 20 g portions placed on top of Baermann funnels. In a mist chamber apparatus, the roots were intermittently sprayed with water at 27 °C for five days. After this, the extracts were collected and *P. vulnus* were identified and counted [12]. Nematode numbers were recorded on a per 1 g basis. In each dormant season, the height of each of the trees in these nematode field screens was measured from the ground level perpendicularly to the maximum height of the trees with an extended ruler. Thus, the tree height was measured under nematode-infested conditions.

The phenotypes of the seven hybrids are shown in Figure S1. Note that phenotypic data for STJM-4 and JMS-12 are missing for *P. vulnus* and vigor. These phenotypes were determined prior to this study during the publication of [19]. Therefore, the hybrids used in this study were unchallenged at the time of RNA extraction.

### 4.3. Sample Collection, RNA Isolation and Sequencing

Healthy white actively growing uninoculated root samples were collected from six plants of each genotype. We consider these “sub replicates” replicates, given that each replicate serves to assess the variation in transcription within each genotype due to the greenhouse conditions (irrigation, humidity, temperature, handling, etc.). The statistical handling of this replication is described in the differential expression analysis section. A total of 42 root samples were chosen for RNA extraction. All samples were collected in the lab and flash-frozen in liquid nitrogen immediately after harvesting. Collected samples were stored at −80 °C before the analysis. For transcriptome analysis by deep sequencing, tissue samples were ground to a fine powder in liquid nitrogen. Total RNA was extracted using the MagMAX™ mirVana™ total RNA isolation kit (Thermo Fisher Scientific, Riverside, CA, USA). Library preparation and transcriptome analysis were carried out by Seqmatic LLC (Fremont, CA, USA). At Seqmatic, RNA library preparation was conducted using an Illumina Stranded mRNA library preparation kit, and transcriptome analysis was carried out using NextSeq High Output Run (Paired-End Read 2 × 150 bp). The raw fastq files were deposited in SRA under BioProject PRJNA726720.

### 4.4. Bioinformatics

Raw fastq files from Seqmatic were preprocessed using an HTStream (v1.3.2) pipeline, for which the source code can be found at https://github.com/s4hts/HTStream (accessed on 6 July 2023). This included hts_SeqScreener to remove PhiX and rRNA sequences, hts_SuperDeduper to remove PCR duplicates, hts_AdapterTrimmer to trim adapters, hts_PolyATTrim to trim poly-A and poly-T sequences from the ends of reads, hts_NTrimmer which trims reads to the longest subsequence that contains no N characters, hts_QWindowTrim to remove low-quality ends of the reads and hts_LengthFilter to remove reads less than 50 bp in length. The details of the preprocessing results can be found in File S7. The sequences used as a reference for rRNA removal were obtained by downloading all predicted rRNAs of the *J. microcarpa* and *J. regia* genomes from NCBIProcessed fastq files were then aligned to a combined genome reference of *J. microcarpa* and *J. regia*. Both the alignment and expression estimation were performed using STAR (v2.7.9a) [47]. The scripts for read processing and alignment can be found at https://github.com/hsaxe/SCRI_ROOT_bash (accessed 6 July 2023).

### 4.5. Splicing Analysis

STAR log.final.out files for each sample from the alignment step were combined using bash commands. These files contain information on AT/AC, GC/AG, GT/AG, total, non-canonical or annotated (sjdb) splices. The splicing information was then analyzed using an R script. The number of splices from each sample was averaged by hybrid and then analyzed for Pearson correlation with the phenotypic data. Only *p*-values of less than 0.05 were considered significant.

### 4.6. Differential Expression Analysis

The gene counts were analyzed using the R programming language for statistical computing and graphics version 4.1.2 in R studio [48,49]. For PCA, all gene counts were normalized by the counts per million mapped reads (CPM) method and any gene with a maximum expression under 30 CPM was excluded from the downstream analysis. Principal component analysis (PCA) was conducted on the filtered expression matrix and the principal component (PC) scores were averaged for each hybrid and used for regression against each trait (Figure 1). A linear model was fit to each gene in the filtered expression matrix for *A. tumefaciens* (crown gall score), *P. cinnamomi* and *P. pini* (average percent root and crown rot), *P. vulnus* (root-lesion nematode count per gram of root) and tree height at three years of age. To help reduce type I error, limma-voom and empirical Bayes smoothing of standard errors were employed. Moreover, to account for pseudoreplication, the biological replicates within each hybrid were treated as random effects within the model. After modeling, genes were considered differentially expressed if their false discovery rate (FDR) was less than or equal to 0.05.

### 4.7. Expression by Haplotype

We used Fisher’s exact test in R to analyze the differences in the number of DEGs between the *J. microcarpa* and *J. regia* haplotypes. Unique lists of DEGs positively and negatively correlated with each trait were extracted from the DEA and used to make a two-by-two contingency matrix. This matrix was used to test the alternative hypothesis that the odds ratio of up genes to down genes between *J. microcarpa* and *J. regia* was not equal to one. An example of the contingency matrix is in Figure S2.

### 4.8. Gene Ontology Analysis

Unique lists of DEGs with their fold changes were extracted from the DEA and used as inputs for the Kolmogorov–Smirnov test to test for coordinated shifts in gene expression by biological process as is used in the PANTHER enrichment test [50,51]. *Juglans regia* was used as the species from which to derive the annotation set for this analysis. *Juglans regia* genes were inferred from *Juglans microcarpa* genes using BLAST (basic local alignment search tool). The GO biological process complete was used as the annotation data set and only terms with an FDR of less than 0.05 were considered enriched. In Figure 3, Figure 4 and Figure 5, the term labels are biased and limited to those terms mentioning “RNA”, “cell wall”, “polysaccharide”, “cellulose”, “glucan”, “defense”, “jasmonic acid”, “abscisic acid”, “salicylic acid” or “ethylene” and an FDR of less than or equal to 0.05. This was performed for two reasons: (i) The results are so large that visualizing and reporting all of them is unreasonable. Therefore, we looked for the biggest patterns in the results and pursued those. (ii) We were able to make a priori hypotheses about certain biological processes of interest and report on those results.

### 4.9. Subcellular Localization Analysis

Unlike the GO BP analysis, we did not need to infer *J. microcarpa* and *J. regia* genes from genes with BLAST. Both the *J. microcarpa* (https://ftp.ncbi.nlm.nih.gov/genomes/all/GCF/004/785/595/GCF_004785595.1_Jm3101_v1.0/GCF_004785595.1_Jm3101_v1.0_protein.faa.gz, accessed 6 July 2023) and *J. regia* (https://ftp.ncbi.nlm.nih.gov/genomes/all/GCF/001/411/555/GCF_001411555.2_Walnut_2.0/GCF_001411555.2_Walnut_2.0_protein.faa.gz, accessed 6 July 2023) proteomes were sent to Dr. Castrense Savojardo at the Bologna Biocomputing Group for subcellular localization prediction using the Bologna Unified Subcellular Component Annotator (BUSCA) [52]. These predictions were used to annotate the DEGs for each trait. Again, unique lists of DEGs with their fold changes were extracted from the DEA and used as inputs for the Kolmogorov–Smirnov test to test for coordinated shifts in gene expression by subcellular localization as is used in the PANTHER enrichment test [50,51]. Only terms with an FDR of less than 0.05 were considered enriched.

### 4.10. R Packages and Code Availability

The R packages Limma [53], edgeR [54], base [48], data.table [55], tidytable [56], dplyr [57], stats [48], ggpubr [58], ggplot2 [59], graphics [48], sjPlot [60], tibble [61], utils [48], tidyr [62], gridExtra [63], OmicsAnalyst [64], tidytext [65], stringr [66], knitr [67] and rmarkdown [68] were used to facilitate all analyses performed in R version 4.1.2. All code used for the differential expression analysis, gene ontology analysis, expression by haplotype analysis and subcellular localization analysis can be found at https://github.com/hsaxe/SCRI_ROOT_R, accessed 6 July 2023.

## 5. Conclusions

This study provided a starting point for functional genomic insights on potential host mechanisms of resistance and susceptibility to *A. tumefaciens*, *Phytophthora* spp. and vigor in *J. microcarpa* × *J. regia* hybrids. Principal component analysis identified some transcriptional repertoires associated with each trait, highlighting the ability of RNA-seq to capture genetic variation related to the traits of interest. This study focused on cell wall biogenesis and cellular barriers. However, other biological processes and subcellular loci were significant in pathogenesis or vigor, such as jasmonic acid, ethylene, abscisic acid, the nucleus and long non-coding RNAs. Moreover, we point out quality candidate genes that are likely to be causal in resistance to *A. tumefaciens* as they are differentially expressed as resistance genes and within the QTL region for resistance reported in a previous QTL study [19]. Current breeding efforts seem well suited to focus on black walnut species because *J. regia* may be a source of susceptibility to *A. tumefaciens* and *Phytophthora* spp. However, *J. microcarpa* may be a source of low vigor. Therefore, careful consideration of the potential tradeoff for desired agronomic traits is suggested when determining the usefulness of these hybrids in future breeding. Aside from *P. vulnus*, the transcripts positively correlated with all traits were enriched in the GO terms of cell wall organization/biogenesis. Moreover, these same genes targeted the cellular barriers significantly more than the transcripts negatively correlated with these traits. These results suggested that increased activity in gene expression related to cell wall biogenesis may be a susceptibility factor in these diseases and a factor promoting vigor in these hybrids. Therefore, modulating cell wall biogenesis may modulate pathogenesis and vigor in walnuts. This new hypothesis must be validated by knocking out genes involved in cell wall biogenesis, such as the cellulose synthases or arabinogalactan proteins. Alternatively, these genes could be inhibited via isoxaben for the cellulose synthases or the Yariv reagent for the arabinogalactan proteins [42,69].

## Figures and Tables

**Table 1:** Correlation of disease traits against principal components of variation from PCA of RNA data. Correlation coefficient and *p*-value are displayed in the plot. CG_Avg. = *A. tumefaciens* phenotypic response, Height_3Y = tree height at three years of age, PHY_Avg = *Phytophthora* spp. phenotypic response, RLN_3Y = *P. vulnus* phenotypic response.

Cor	PC	*p*-Value	Trait
−0.804	PC2	2.94 × 10^−2^	CG
−0.639	PC5	1.22 × 10^−1^	PHY
−0.698	PC4	1.90 × 10^−1^	RLN_3Y
−0.867	PC1	5.69 × 10^−2^	length_3Y

**Table 2:** Summary of the results of the differential expression analysis. CG_Avg. = *A. tumefaciens* phenotypic response, Height_3Y = tree height at three years of age, PHY_Avg = *Phytophthora* spp. phenotypic response, RLN_3Y = *P. vulnus* phenotypic response.

Statistic	CG_Avg	PHY_Avg	RLN_3Y	Height _3Y
Down	3888	1061	13	3381
NotSig	13,290	17,731	17,962	12,658
Up	2021	407	7	1943

**Table 3:** DEGs observed within QTL region of chromosome 4D from [19].

Gene ID	Protein Product	Trait	logFC	Start	End
121260033	small nuclear ribonucleoprotein SmD3b	CG	−0.647	26,403,410	26,405,628
121260019	dolichol-phosphate mannosyltransferase subunit 1 isoform X1	CG	−0.346	26,421,312	26,423,874
121259960	pre-rRNA-processing protein TSR1 homolog	CG	−0.295	26,449,293	26,457,407
121260033	small nuclear ribonucleoprotein SmD3b	Height_3Y	−0.530	26,403,410	26,405,628
121259974	probable acyl-activating enzyme 1, peroxisomal	Height_3Y	−0.512	26,477,730	26,481,055
121259960	pre-rRNA-processing protein TSR1 homolog	Height_3Y	−0.256	26,449,293	26,457,407

**Table 4:** Correlation analysis of traits analyzed in this study. CG_Avg. = *A. tumefaciens* phenotypic response, Height_3Y = tree height at three years of age, PHY_Avg = *Phytophthora* spp. phenotypic response, RLN_3Y = *P. vulnus* phenotypic response.

Relationship		Cor	*p*-Value
Height _3Y	PHY_Avg	0.983	0.003
Height _3Y	CG_Avg	0.936	0.019
PHY_Avg	CG_Avg	0.705	0.077
RLN_3Y	PHY_Avg	0.663	0.222
RLN_3Y	Height _3Y	0.574	0.311
RLN_3Y	CG_Avg	0.547	0.341

## Data Availability

Data is contained within the article and Supplementary Materials.

## Supplementary Materials

The following supporting information can be downloaded at: https://www.mdpi.com/article/10.3390/ijms25020931/s1.
