# Supplementary material for: Transcriptomic Evidence of a Link between Cell Wall Biogenesis, Pathogenesis, and Vigor in Walnut Root and Trunk Diseases
Source: Int J Mol Sci. 2024 Jan 11;25(2):931. doi: 10.3390/ijms25020931 (PMC10815794; doi:10.3390/ijms25020931)
Supplement: Supplementary file 1 [file ijms-25-00931-s001.zip › File S7.html]

SCRI\_ROOT\_RNAseq\_MultiQC\_HTS\_STAR\_Report\_Jm3101\_v1.0\_Walnut\_2.0: MultiQC Report


# Toggle navigation v1.10.dev0

# SCRI\_ROOT\_RNAseq\_MultiQC\_HTS\_STAR\_Report\_Jm3101\_v1.0\_Walnut\_2.0

Loading report..

- General Stats
- STAR
  - Alignment Scores
  - Gene Counts
- HTStream
  - hts\_Stats
  - hts\_SeqScreener
  - hts\_SeqScreener 2
  - hts\_SuperDeduper
  - hts\_AdapterTrimmer
  - hts\_PolyATTrim
  - hts\_NTrimmer
  - hts\_QWindowTrim
  - hts\_LengthFilter
  - hts\_Stats 2

Toolbox

### MultiQC Toolbox

#### Apply Highlight Samples

+

Regex mode off
help
 Clear

#### Apply Rename Samples

+

Click here for bulk input.

Paste two columns of a tab-delimited table here (eg. from Excel).

First column should be the old name, second column the new name.

Add

Regex mode off
help
 Clear

#### Apply Show / Hide Samples

Hide matching samples

Show only matching samples

+

Regex mode off
help
 Clear

#### Export Plots

- Images
- Data

px

px

Aspect ratio

PNG
JPEG
SVG

Plot scaling

X

Download the raw data used to create the plots in this report below:

Format:

Tab-separated
Comma-separated
JSON

Note that additional data was saved in `SCRI_ROOT_RNAseq_MultiQC_HTS_STAR_Report_Jm3101_v1.0_Walnut_2.0_multiqc_report_data` when this report was generated.

---

##### Choose Plots

 All
 None

---


   Download Plot Images

If you use plots from MultiQC in a publication or presentation, please cite:

> **MultiQC: Summarize analysis results for multiple tools and samples in a single report**  
> *Philip Ewels, Måns Magnusson, Sverker Lundin and Max Käller*  
> Bioinformatics (2016)  
> doi: 10.1093/bioinformatics/btw354  
> PMID: 27312411

#### Save Settings

You can save the toolbox settings for this report to the browser.

 Save


---

#### Load Settings

Choose a saved report profile from the dropdown box below:

[ select ]

Load
 Delete
 Set default
 Clear default

#### About MultiQC

This report was generated using MultiQC, version 1.10.dev0

You can see a YouTube video describing how to use MultiQC reports here:
https://youtu.be/qPbIlO\_KWN0

For more information about MultiQC, including other videos and
extensive documentation, please visit http://multiqc.info

You can report bugs, suggest improvements and find the source code for MultiQC on GitHub:
https://github.com/ewels/MultiQC

MultiQC is published in Bioinformatics:

> **MultiQC: Summarize analysis results for multiple tools and samples in a single report**  
> *Philip Ewels, Måns Magnusson, Sverker Lundin and Max Käller*  
> Bioinformatics (2016)  
> doi: 10.1093/bioinformatics/btw354  
> PMID: 27312411

# 

# SCRI\_ROOT\_RNAseq\_MultiQC\_HTS\_STAR\_Report\_Jm3101\_v1.0\_Walnut\_2.0

A modular tool to aggregate results from bioinformatics analyses across many samples into a single report.

#### JavaScript Disabled

MultiQC reports use JavaScript for plots and toolbox functions. It looks like
you have JavaScript disabled in your web browser. Please note that many of the report
functions will not work as intended.

Loading report..

Report
generated on 2023-12-23, 14:57
based on data in:

- `/share/dandekarlab/Houston/SCRI_Root_RNAseq_work/01-HTS_Preproc`
- `/share/dandekarlab/Houston/SCRI_Root_RNAseq_work/05-STAR_alignment_2pass_combined_genomes`

---

×
don't show again

**Welcome!** Not sure where to start?  
Watch a tutorial video
  *(6:06)*

## General Statistics

 Copy table

 Configure Columns

 Sort by highlight

 Plot
Showing 84/84 rows and 2/2 columns.

| Sample Name | % Aligned | M Aligned |
| --- | --- | --- |
| 29JM-11-1 | 76.3% | 2.5 |
| 29JM-11-1\_\_STARpass1 | 76.3% | 2.5 |
| 29JM-11-2 | 67.6% | 0.8 |
| 29JM-11-2\_\_STARpass1 | 67.5% | 0.8 |
| 29JM-11-3 | 77.7% | 3.0 |
| 29JM-11-3\_\_STARpass1 | 77.8% | 3.0 |
| 29JM-11-4 | 81.0% | 5.2 |
| 29JM-11-4\_\_STARpass1 | 81.0% | 5.3 |
| 29JM-11-5 | 81.3% | 6.2 |
| 29JM-11-5\_\_STARpass1 | 81.4% | 6.2 |
| 29JM-11-6 | 86.5% | 7.5 |
| 29JM-11-6\_\_STARpass1 | 86.7% | 7.5 |
| JMS-12-1 | 85.7% | 4.8 |
| JMS-12-1\_\_STARpass1 | 85.8% | 4.9 |
| JMS-12-2 | 85.4% | 4.5 |
| JMS-12-2\_\_STARpass1 | 85.6% | 4.5 |
| JMS-12-3 | 84.4% | 5.6 |
| JMS-12-3\_\_STARpass1 | 84.5% | 5.6 |
| JMS-12-4 | 84.0% | 4.3 |
| JMS-12-4\_\_STARpass1 | 84.1% | 4.3 |
| JMS-12-5 | 84.2% | 5.7 |
| JMS-12-5\_\_STARpass1 | 84.4% | 5.7 |
| JMS-12-6 | 87.0% | 7.3 |
| JMS-12-6\_\_STARpass1 | 87.1% | 7.3 |
| MS1-122-1 | 86.0% | 4.9 |
| MS1-122-1\_\_STARpass1 | 86.1% | 4.9 |
| MS1-122-2 | 86.9% | 6.7 |
| MS1-122-2\_\_STARpass1 | 87.0% | 6.7 |
| MS1-122-3 | 88.5% | 7.3 |
| MS1-122-3\_\_STARpass1 | 88.6% | 7.3 |
| MS1-122-4 | 79.7% | 3.9 |
| MS1-122-4\_\_STARpass1 | 79.7% | 3.9 |
| MS1-122-5 | 88.0% | 9.9 |
| MS1-122-5\_\_STARpass1 | 88.1% | 9.9 |
| MS1-122-6 | 77.9% | 4.0 |
| MS1-122-6\_\_STARpass1 | 77.9% | 4.0 |
| MS1-36-1 | 87.7% | 7.9 |
| MS1-36-1\_\_STARpass1 | 88.0% | 7.9 |
| MS1-36-2 | 82.7% | 5.3 |
| MS1-36-2\_\_STARpass1 | 83.0% | 5.4 |
| MS1-36-3 | 89.0% | 12.7 |
| MS1-36-3\_\_STARpass1 | 89.2% | 12.7 |
| MS1-36-4 | 85.5% | 5.9 |
| MS1-36-4\_\_STARpass1 | 85.8% | 5.9 |
| MS1-36-5 | 84.1% | 4.1 |
| MS1-36-5\_\_STARpass1 | 84.4% | 4.1 |
| MS1-36-6 | 83.6% | 7.7 |
| MS1-36-6\_\_STARpass1 | 83.8% | 7.8 |
| MS1-41-1 | 78.6% | 5.7 |
| MS1-41-1\_\_STARpass1 | 78.7% | 5.7 |
| MS1-41-2 | 77.9% | 3.3 |
| MS1-41-2\_\_STARpass1 | 78.0% | 3.3 |
| MS1-41-3 | 86.7% | 5.1 |
| MS1-41-3\_\_STARpass1 | 86.8% | 5.1 |
| MS1-41-4 | 86.5% | 4.6 |
| MS1-41-4\_\_STARpass1 | 86.7% | 4.6 |
| MS1-41-5 | 80.0% | 6.0 |
| MS1-41-5\_\_STARpass1 | 80.1% | 6.0 |
| MS1-41-6 | 83.9% | 5.0 |
| MS1-41-6\_\_STARpass1 | 84.1% | 5.0 |
| MS1-56-1 | 86.2% | 6.1 |
| MS1-56-1\_\_STARpass1 | 86.5% | 6.1 |
| MS1-56-2 | 85.8% | 5.7 |
| MS1-56-2\_\_STARpass1 | 86.1% | 5.7 |
| MS1-56-3 | 83.8% | 5.9 |
| MS1-56-3\_\_STARpass1 | 84.0% | 5.9 |
| MS1-56-4 | 85.1% | 4.7 |
| MS1-56-4\_\_STARpass1 | 85.3% | 4.7 |
| MS1-56-5 | 60.3% | 1.6 |
| MS1-56-5\_\_STARpass1 | 60.1% | 1.6 |
| MS1-56-6 | 84.6% | 7.8 |
| MS1-56-6\_\_STARpass1 | 84.9% | 7.8 |
| STJM-4-1 | 59.0% | 0.7 |
| STJM-4-1\_\_STARpass1 | 59.0% | 0.7 |
| STJM-4-2 | 81.7% | 2.3 |
| STJM-4-2\_\_STARpass1 | 82.0% | 2.3 |
| STJM-4-3 | 82.5% | 1.3 |
| STJM-4-3\_\_STARpass1 | 82.8% | 1.4 |
| STJM-4-4 | 83.5% | 3.3 |
| STJM-4-4\_\_STARpass1 | 83.7% | 3.3 |
| STJM-4-5 | 78.9% | 1.5 |
| STJM-4-5\_\_STARpass1 | 79.1% | 1.5 |
| STJM-4-6 | 77.9% | 1.8 |
| STJM-4-6\_\_STARpass1 | 77.9% | 1.8 |

×

#### General Statistics: Columns

Uncheck the tick box to hide columns. Click and drag the handle on the left to change order.

Show All
Show None

| Sort | Visible | Group | Column | Description | ID | Scale |
| --- | --- | --- | --- | --- | --- | --- |
| || |  | STAR | % Aligned | % Uniquely mapped reads | `uniquely_mapped_percent` | None |
| || |  | STAR | M Aligned | Uniquely mapped reads (millions) | `uniquely_mapped` | read\_count |

Close

## STAR

STAR is an ultrafast universal RNA-seq aligner.

### Alignment Scores

Number of Reads
Percentages

loading..

---

### Gene Counts

Statistics from results generated using `--quantMode GeneCounts`. The three tabs show counts for unstranded RNA-seq, counts for the 1st read strand aligned with RNA and counts for the 2nd read strand aligned with RNA.

Number of Reads
Percentages

Unstranded
Same Stranded
Reverse Stranded

loading..

---

## HTStream

HTStream quality control and processing pipeline for High Throughput Sequencing data.

{"htstream\_number\_of\_samples": 42}

### hts\_Stats

Generates a JSON formatted file containing a set of statistical measures about the input read data.

 Copy table

 Configure Columns

 Sort by highlight

 Plot
Showing 42/42 rows and 6/6 columns.

| Sample Name | % PE | % R1 Q30 | % R2 Q30 | GC Content | N Content | Notes |
| --- | --- | --- | --- | --- | --- | --- |
| 29JM-11-1 | 100.00% | 89.95% | 86.19% | 48.78% | 0.0000% | initial stats |
| 29JM-11-2 | 100.00% | 86.87% | 84.50% | 50.27% | 0.0000% | initial stats |
| 29JM-11-3 | 100.00% | 89.06% | 86.00% | 48.08% | 0.0000% | initial stats |
| 29JM-11-4 | 100.00% | 89.58% | 86.71% | 47.69% | 0.0000% | initial stats |
| 29JM-11-5 | 100.00% | 89.44% | 86.47% | 48.44% | 0.0000% | initial stats |
| 29JM-11-6 | 100.00% | 90.53% | 86.97% | 46.47% | 0.0000% | initial stats |
| JMS-12-1 | 100.00% | 90.14% | 86.46% | 46.18% | 0.0000% | initial stats |
| JMS-12-2 | 100.00% | 90.18% | 86.28% | 46.62% | 0.0000% | initial stats |
| JMS-12-3 | 100.00% | 90.60% | 86.83% | 46.27% | 0.0000% | initial stats |
| JMS-12-4 | 100.00% | 89.14% | 86.58% | 48.52% | 0.0000% | initial stats |
| JMS-12-5 | 100.00% | 90.15% | 86.38% | 46.36% | 0.0000% | initial stats |
| JMS-12-6 | 100.00% | 90.36% | 86.67% | 46.17% | 0.0000% | initial stats |
| MS1-122-1 | 100.00% | 89.93% | 84.79% | 45.76% | 0.0000% | initial stats |
| MS1-122-2 | 100.00% | 89.44% | 86.22% | 46.30% | 0.0000% | initial stats |
| MS1-122-3 | 100.00% | 89.34% | 83.76% | 46.41% | 0.0000% | initial stats |
| MS1-122-4 | 100.00% | 89.14% | 84.43% | 47.69% | 0.0000% | initial stats |
| MS1-122-5 | 100.00% | 89.41% | 85.95% | 46.18% | 0.0000% | initial stats |
| MS1-122-6 | 100.00% | 89.58% | 86.75% | 47.98% | 0.0000% | initial stats |
| MS1-36-1 | 100.00% | 89.76% | 85.56% | 46.47% | 0.0000% | initial stats |
| MS1-36-2 | 100.00% | 89.54% | 82.48% | 46.62% | 0.0000% | initial stats |
| MS1-36-3 | 100.00% | 90.71% | 85.49% | 45.79% | 0.0000% | initial stats |
| MS1-36-4 | 100.00% | 90.80% | 87.34% | 46.18% | 0.0000% | initial stats |
| MS1-36-5 | 100.00% | 88.71% | 83.98% | 47.56% | 0.0000% | initial stats |
| MS1-36-6 | 100.00% | 90.71% | 86.18% | 47.12% | 0.0000% | initial stats |
| MS1-41-1 | 100.00% | 89.81% | 86.50% | 46.46% | 0.0000% | initial stats |
| MS1-41-2 | 100.00% | 88.67% | 85.74% | 49.03% | 0.0000% | initial stats |
| MS1-41-3 | 100.00% | 90.09% | 85.91% | 46.46% | 0.0000% | initial stats |
| MS1-41-4 | 100.00% | 89.72% | 85.65% | 46.91% | 0.0000% | initial stats |
| MS1-41-5 | 100.00% | 89.73% | 86.31% | 49.19% | 0.0000% | initial stats |
| MS1-41-6 | 100.00% | 89.41% | 85.96% | 47.14% | 0.0000% | initial stats |
| MS1-56-1 | 100.00% | 89.40% | 86.18% | 46.52% | 0.0000% | initial stats |
| MS1-56-2 | 100.00% | 89.40% | 85.97% | 47.35% | 0.0000% | initial stats |
| MS1-56-3 | 100.00% | 89.78% | 86.63% | 47.04% | 0.0000% | initial stats |
| MS1-56-4 | 100.00% | 90.23% | 87.04% | 47.05% | 0.0000% | initial stats |
| MS1-56-5 | 100.00% | 89.01% | 86.58% | 51.90% | 0.0000% | initial stats |
| MS1-56-6 | 100.00% | 90.49% | 87.13% | 47.99% | 0.0000% | initial stats |
| STJM-4-1 | 100.00% | 86.14% | 85.65% | 52.02% | 0.0000% | initial stats |
| STJM-4-2 | 100.00% | 88.29% | 86.19% | 47.83% | 0.0000% | initial stats |
| STJM-4-3 | 100.00% | 88.24% | 85.31% | 46.71% | 0.0000% | initial stats |
| STJM-4-4 | 100.00% | 89.92% | 86.18% | 47.84% | 0.0000% | initial stats |
| STJM-4-5 | 100.00% | 87.84% | 85.25% | 48.26% | 0.0000% | initial stats |
| STJM-4-6 | 100.00% | 89.11% | 85.65% | 48.27% | 0.0000% | initial stats |

×

#### Table Qejo: Columns

Uncheck the tick box to hide columns. Click and drag the handle on the left to change order.

Show All
Show None

| Sort | Visible | Group | Column | Description | ID | Scale |
| --- | --- | --- | --- | --- | --- | --- |
| || |  | % PE | % PE | percentage of paried end reads | `St_PE_Fraction1` | None |
| || |  | % R1 Q30 | % R1 Q30 | percentage of read 1 bps Q30 or greater | `St_R1_Q301` | None |
| || |  | % R2 Q30 | % R2 Q30 | percentage of read 2 bps Q30 or greater | `St_R2_Q301` | None |
| || |  | GC Content | GC Content | Percentage of bps that are G or C | `St_GC_Content1` | None |
| || |  | N Content | N Content | Percentage of bps that are N | `St_N_Content1` | None |
| || |  | Notes | Notes | Notes | `St_Notes1` | None |

Close

  

#### Read Lengths: Paired End

Distribution of read lengths for each sample.

**Notice:** Each sample has a uniform read length distribution.

 Copy table

 Configure Columns

 Sort by highlight

 Plot
Showing 42/42 rows and 2/2 columns.

| Sample Name | R1 Read Lengths | R2 Read Lengths |
| --- | --- | --- |
| 29JM-11-1 | 151 | 151 |
| 29JM-11-2 | 151 | 151 |
| 29JM-11-3 | 151 | 151 |
| 29JM-11-4 | 151 | 151 |
| 29JM-11-5 | 151 | 151 |
| 29JM-11-6 | 151 | 151 |
| JMS-12-1 | 151 | 151 |
| JMS-12-2 | 151 | 151 |
| JMS-12-3 | 151 | 151 |
| JMS-12-4 | 151 | 151 |
| JMS-12-5 | 151 | 151 |
| JMS-12-6 | 151 | 151 |
| MS1-122-1 | 151 | 151 |
| MS1-122-2 | 151 | 151 |
| MS1-122-3 | 151 | 151 |
| MS1-122-4 | 151 | 151 |
| MS1-122-5 | 151 | 151 |
| MS1-122-6 | 151 | 151 |
| MS1-36-1 | 151 | 151 |
| MS1-36-2 | 151 | 151 |
| MS1-36-3 | 151 | 151 |
| MS1-36-4 | 151 | 151 |
| MS1-36-5 | 151 | 151 |
| MS1-36-6 | 151 | 151 |
| MS1-41-1 | 151 | 151 |
| MS1-41-2 | 151 | 151 |
| MS1-41-3 | 151 | 151 |
| MS1-41-4 | 151 | 151 |
| MS1-41-5 | 151 | 151 |
| MS1-41-6 | 151 | 151 |
| MS1-56-1 | 151 | 151 |
| MS1-56-2 | 151 | 151 |
| MS1-56-3 | 151 | 151 |
| MS1-56-4 | 151 | 151 |
| MS1-56-5 | 151 | 151 |
| MS1-56-6 | 151 | 151 |
| STJM-4-1 | 151 | 151 |
| STJM-4-2 | 151 | 151 |
| STJM-4-3 | 151 | 151 |
| STJM-4-4 | 151 | 151 |
| STJM-4-5 | 151 | 151 |
| STJM-4-6 | 151 | 151 |

×

#### Table Uydh: Columns

Uncheck the tick box to hide columns. Click and drag the handle on the left to change order.

Show All
Show None

| Sort | Visible | Group | Column | Description | ID | Scale |
| --- | --- | --- | --- | --- | --- | --- |
| || |  | R1 Read Lengths | R1 Read Lengths | Length of R1 reads (uniform for each sample). | `St_Read_Lengths_R1_3272621945207` | None |
| || |  | R2 Read Lengths | R2 Read Lengths | Length of R2 reads (uniform for each sample). | `St_Read_Lengths_R2_3272621945207` | None |

Close

  

#### Base by Cycle: Paired End

Provides a measure of the uniformity of a distribution. The higher the average is at a certain position,
the more unequal the base pair composition. N's are excluded from this calculation.

Avg. Diff. from 25%
Base by Cycle

loading..

29JM-11-1
29JM-11-2
29JM-11-3
29JM-11-4
29JM-11-5
29JM-11-6
JMS-12-1
JMS-12-2
JMS-12-3
JMS-12-4
JMS-12-5
JMS-12-6
MS1-122-1
MS1-122-2
MS1-122-3
MS1-122-4
MS1-122-5
MS1-122-6
MS1-36-1
MS1-36-2
MS1-36-3
MS1-36-4
MS1-36-5
MS1-36-6
MS1-41-1
MS1-41-2
MS1-41-3
MS1-41-4
MS1-41-5
MS1-41-6
MS1-56-1
MS1-56-2
MS1-56-3
MS1-56-4
MS1-56-5
MS1-56-6
STJM-4-1
STJM-4-2
STJM-4-3
STJM-4-4
STJM-4-5
STJM-4-6

loading..

  

#### Quality by Cycle: Paired End

Mean quality score for each position along the read.
Sample is colored red if less than 60% of bps have mean score of at least Q30,
orange if between 60% and 80%, and green otherwise.

Mean Quality
Quality by Cycle

loading..

29JM-11-1
29JM-11-2
29JM-11-3
29JM-11-4
29JM-11-5
29JM-11-6
JMS-12-1
JMS-12-2
JMS-12-3
JMS-12-4
JMS-12-5
JMS-12-6
MS1-122-1
MS1-122-2
MS1-122-3
MS1-122-4
MS1-122-5
MS1-122-6
MS1-36-1
MS1-36-2
MS1-36-3
MS1-36-4
MS1-36-5
MS1-36-6
MS1-41-1
MS1-41-2
MS1-41-3
MS1-41-4
MS1-41-5
MS1-41-6
MS1-56-1
MS1-56-2
MS1-56-3
MS1-56-4
MS1-56-5
MS1-56-6
STJM-4-1
STJM-4-2
STJM-4-3
STJM-4-4
STJM-4-5
STJM-4-6

Sort by highlight

loading..

---

### hts\_SeqScreener

A simple sequence screening tool which uses a kmer lookup approach to identify reads from an unwanted source.

 Copy table

 Configure Columns

 Sort by highlight

 Plot
Showing 42/42 rows and 3/3 columns.

| Sample Name | PE hits | % PE Hits | Notes |
| --- | --- | --- | --- |
| 29JM-11-1 | 1 | 0.0000% | screen phix |
| 29JM-11-2 | 0 | 0.0000% | screen phix |
| 29JM-11-3 | 0 | 0.0000% | screen phix |
| 29JM-11-4 | 0 | 0.0000% | screen phix |
| 29JM-11-5 | 0 | 0.0000% | screen phix |
| 29JM-11-6 | 0 | 0.0000% | screen phix |
| JMS-12-1 | 0 | 0.0000% | screen phix |
| JMS-12-2 | 2 | 0.0000% | screen phix |
| JMS-12-3 | 0 | 0.0000% | screen phix |
| JMS-12-4 | 1 | 0.0000% | screen phix |
| JMS-12-5 | 3 | 0.0000% | screen phix |
| JMS-12-6 | 1 | 0.0000% | screen phix |
| MS1-122-1 | 0 | 0.0000% | screen phix |
| MS1-122-2 | 0 | 0.0000% | screen phix |
| MS1-122-3 | 0 | 0.0000% | screen phix |
| MS1-122-4 | 0 | 0.0000% | screen phix |
| MS1-122-5 | 0 | 0.0000% | screen phix |
| MS1-122-6 | 2 | 0.0000% | screen phix |
| MS1-36-1 | 0 | 0.0000% | screen phix |
| MS1-36-2 | 0 | 0.0000% | screen phix |
| MS1-36-3 | 0 | 0.0000% | screen phix |
| MS1-36-4 | 1 | 0.0000% | screen phix |
| MS1-36-5 | 0 | 0.0000% | screen phix |
| MS1-36-6 | 0 | 0.0000% | screen phix |
| MS1-41-1 | 0 | 0.0000% | screen phix |
| MS1-41-2 | 0 | 0.0000% | screen phix |
| MS1-41-3 | 0 | 0.0000% | screen phix |
| MS1-41-4 | 0 | 0.0000% | screen phix |
| MS1-41-5 | 0 | 0.0000% | screen phix |
| MS1-41-6 | 1 | 0.0000% | screen phix |
| MS1-56-1 | 0 | 0.0000% | screen phix |
| MS1-56-2 | 0 | 0.0000% | screen phix |
| MS1-56-3 | 0 | 0.0000% | screen phix |
| MS1-56-4 | 2 | 0.0000% | screen phix |
| MS1-56-5 | 1 | 0.0000% | screen phix |
| MS1-56-6 | 1 | 0.0000% | screen phix |
| STJM-4-1 | 0 | 0.0000% | screen phix |
| STJM-4-2 | 1 | 0.0000% | screen phix |
| STJM-4-3 | 0 | 0.0000% | screen phix |
| STJM-4-4 | 3 | 0.0000% | screen phix |
| STJM-4-5 | 0 | 0.0000% | screen phix |
| STJM-4-6 | 0 | 0.0000% | screen phix |

×

#### Table Fojq: Columns

Uncheck the tick box to hide columns. Click and drag the handle on the left to change order.

Show All
Show None

| Sort | Visible | Group | Column | Description | ID | Scale |
| --- | --- | --- | --- | --- | --- | --- |
| || |  | PE hits | PE hits | Number of Paired End Reads with Sequence | `Ss_PE_hits1` | None |
| || |  | % PE Lost | % PE Hits | Percentage of Paired End Reads Lost | `Ss_PE_%_hits1` | None |
| || |  | Notes | Notes | Notes | `Ss_Notes1` | None |

Close

---

### hts\_SeqScreener 2

A simple sequence screening tool which uses a kmer lookup approach to identify reads from an unwanted source.

 Copy table

 Configure Columns

 Sort by highlight

 Plot
Showing 42/42 rows and 3/3 columns.

| Sample Name | PE hits | % PE Hits | Notes |
| --- | --- | --- | --- |
| 29JM-11-1 | 3093995 | 21.6919% | count the number of rRNA reads |
| 29JM-11-2 | 4061581 | 41.7823% | count the number of rRNA reads |
| 29JM-11-3 | 2926056 | 24.6395% | count the number of rRNA reads |
| 29JM-11-4 | 3746781 | 20.0501% | count the number of rRNA reads |
| 29JM-11-5 | 4634312 | 27.0060% | count the number of rRNA reads |
| 29JM-11-6 | 239295 | 1.1400% | count the number of rRNA reads |
| JMS-12-1 | 172648 | 1.2984% | count the number of rRNA reads |
| JMS-12-2 | 547911 | 4.9555% | count the number of rRNA reads |
| JMS-12-3 | 808672 | 5.1465% | count the number of rRNA reads |
| JMS-12-4 | 3004810 | 27.8810% | count the number of rRNA reads |
| JMS-12-5 | 692571 | 4.4940% | count the number of rRNA reads |
| JMS-12-6 | 230864 | 1.6761% | count the number of rRNA reads |
| MS1-122-1 | 138010 | 1.2019% | count the number of rRNA reads |
| MS1-122-2 | 548745 | 3.4746% | count the number of rRNA reads |
| MS1-122-3 | 505136 | 3.1594% | count the number of rRNA reads |
| MS1-122-4 | 2801765 | 15.7665% | count the number of rRNA reads |
| MS1-122-5 | 719335 | 3.8210% | count the number of rRNA reads |
| MS1-122-6 | 4411886 | 22.8508% | count the number of rRNA reads |
| MS1-36-1 | 642047 | 3.0498% | count the number of rRNA reads |
| MS1-36-2 | 1477544 | 6.3310% | count the number of rRNA reads |
| MS1-36-3 | 328659 | 1.5200% | count the number of rRNA reads |
| MS1-36-4 | 339714 | 2.6586% | count the number of rRNA reads |
| MS1-36-5 | 1614528 | 11.6100% | count the number of rRNA reads |
| MS1-36-6 | 2975559 | 11.1945% | count the number of rRNA reads |
| MS1-41-1 | 2602501 | 13.4520% | count the number of rRNA reads |
| MS1-41-2 | 3546070 | 27.8019% | count the number of rRNA reads |
| MS1-41-3 | 314666 | 2.9254% | count the number of rRNA reads |
| MS1-41-4 | 395811 | 4.0205% | count the number of rRNA reads |
| MS1-41-5 | 5689857 | 24.8027% | count the number of rRNA reads |
| MS1-41-6 | 1580991 | 10.0976% | count the number of rRNA reads |
| MS1-56-1 | 474936 | 2.6893% | count the number of rRNA reads |
| MS1-56-2 | 1401384 | 8.1975% | count the number of rRNA reads |
| MS1-56-3 | 953551 | 5.8386% | count the number of rRNA reads |
| MS1-56-4 | 866827 | 7.1640% | count the number of rRNA reads |
| MS1-56-5 | 8185566 | 62.0490% | count the number of rRNA reads |
| MS1-56-6 | 3294702 | 13.4853% | count the number of rRNA reads |
| STJM-4-1 | 6421285 | 61.4649% | count the number of rRNA reads |
| STJM-4-2 | 672639 | 4.2700% | count the number of rRNA reads |
| STJM-4-3 | 190321 | 1.9589% | count the number of rRNA reads |
| STJM-4-4 | 1009984 | 7.9049% | count the number of rRNA reads |
| STJM-4-5 | 1374979 | 10.2073% | count the number of rRNA reads |
| STJM-4-6 | 1763251 | 16.5958% | count the number of rRNA reads |

×

#### Table Vkdn: Columns

Uncheck the tick box to hide columns. Click and drag the handle on the left to change order.

Show All
Show None

| Sort | Visible | Group | Column | Description | ID | Scale |
| --- | --- | --- | --- | --- | --- | --- |
| || |  | PE hits | PE hits | Number of Paired End Reads with Sequence | `Ss_PE_hits2` | None |
| || |  | % PE Lost | % PE Hits | Percentage of Paired End Reads Lost | `Ss_PE_%_hits2` | None |
| || |  | Notes | Notes | Notes | `Ss_Notes2` | None |

Close

---

### hts\_SuperDeduper

A reference free duplicate read removal tool.

 Copy table

 Configure Columns

 Sort by highlight

 Plot
Showing 42/42 rows and 3/3 columns.

| Sample Name | % Duplicates | % Ignored | Notes |
| --- | --- | --- | --- |
| 29JM-11-1 | 76.94% | 0.00% | remove PCR duplicates |
| 29JM-11-2 | 88.32% | 0.00% | remove PCR duplicates |
| 29JM-11-3 | 68.00% | 0.00% | remove PCR duplicates |
| 29JM-11-4 | 65.30% | 0.00% | remove PCR duplicates |
| 29JM-11-5 | 55.68% | 0.00% | remove PCR duplicates |
| 29JM-11-6 | 58.92% | 0.00% | remove PCR duplicates |
| JMS-12-1 | 57.40% | 0.00% | remove PCR duplicates |
| JMS-12-2 | 52.06% | 0.00% | remove PCR duplicates |
| JMS-12-3 | 57.80% | 0.00% | remove PCR duplicates |
| JMS-12-4 | 52.85% | 0.00% | remove PCR duplicates |
| JMS-12-5 | 56.14% | 0.00% | remove PCR duplicates |
| JMS-12-6 | 39.00% | 0.00% | remove PCR duplicates |
| MS1-122-1 | 50.35% | 0.00% | remove PCR duplicates |
| MS1-122-2 | 50.99% | 0.00% | remove PCR duplicates |
| MS1-122-3 | 48.44% | 0.00% | remove PCR duplicates |
| MS1-122-4 | 72.74% | 0.00% | remove PCR duplicates |
| MS1-122-5 | 40.25% | 0.00% | remove PCR duplicates |
| MS1-122-6 | 73.14% | 0.00% | remove PCR duplicates |
| MS1-36-1 | 57.28% | 0.00% | remove PCR duplicates |
| MS1-36-2 | 72.32% | 0.00% | remove PCR duplicates |
| MS1-36-3 | 34.10% | 0.00% | remove PCR duplicates |
| MS1-36-4 | 46.29% | 0.00% | remove PCR duplicates |
| MS1-36-5 | 64.75% | 0.00% | remove PCR duplicates |
| MS1-36-6 | 65.12% | 0.00% | remove PCR duplicates |
| MS1-41-1 | 62.24% | 0.00% | remove PCR duplicates |
| MS1-41-2 | 66.74% | 0.00% | remove PCR duplicates |
| MS1-41-3 | 45.55% | 0.00% | remove PCR duplicates |
| MS1-41-4 | 45.78% | 0.00% | remove PCR duplicates |
| MS1-41-5 | 67.23% | 0.00% | remove PCR duplicates |
| MS1-41-6 | 62.07% | 0.00% | remove PCR duplicates |
| MS1-56-1 | 59.95% | 0.00% | remove PCR duplicates |
| MS1-56-2 | 61.34% | 0.00% | remove PCR duplicates |
| MS1-56-3 | 57.05% | 0.00% | remove PCR duplicates |
| MS1-56-4 | 54.76% | 0.00% | remove PCR duplicates |
| MS1-56-5 | 79.39% | 0.00% | remove PCR duplicates |
| MS1-56-6 | 62.21% | 0.00% | remove PCR duplicates |
| STJM-4-1 | 89.31% | 0.00% | remove PCR duplicates |
| STJM-4-2 | 81.81% | 0.00% | remove PCR duplicates |
| STJM-4-3 | 83.16% | 0.00% | remove PCR duplicates |
| STJM-4-4 | 69.37% | 0.00% | remove PCR duplicates |
| STJM-4-5 | 85.72% | 0.00% | remove PCR duplicates |
| STJM-4-6 | 78.60% | 0.00% | remove PCR duplicates |

×

#### Table Isdt: Columns

Uncheck the tick box to hide columns. Click and drag the handle on the left to change order.

Show All
Show None

| Sort | Visible | Group | Column | Description | ID | Scale |
| --- | --- | --- | --- | --- | --- | --- |
| || |  | % Duplicates | % Duplicates | Percentage of Duplicate Reads (SE and PE) | `Sd_%_Duplicates1` | None |
| || |  | % Ignored | % Ignored | Percentage of Ignored Reads (SE and PE) | `Sd_%_Ignored1` | None |
| || |  | Notes | Notes | Notes | `Sd_Notes1` | None |

Close

  

#### SuperDeduper: Duplicate Saturation

Plots the number of duplicates against the number of unique reads per sample.

loading..

---

### hts\_AdapterTrimmer

Trims adapters which are sequenced when the fragment insert length is shorter than the read length.

 Copy table

 Configure Columns

 Sort by highlight

 Plot
Showing 42/42 rows and 4/4 columns.

| Sample Name | % Bp Lost | % Adapters | Avg. Bps Trimmed | Notes |
| --- | --- | --- | --- | --- |
| 29JM-11-1 | 4.99% | 44.71% | 33.67 | trim adapters |
| 29JM-11-2 | 1.92% | 17.47% | 33.10 | trim adapters |
| 29JM-11-3 | 5.35% | 46.86% | 34.47 | trim adapters |
| 29JM-11-4 | 4.87% | 43.24% | 34.02 | trim adapters |
| 29JM-11-5 | 4.89% | 44.18% | 33.41 | trim adapters |
| 29JM-11-6 | 4.57% | 42.20% | 32.69 | trim adapters |
| JMS-12-1 | 5.31% | 47.35% | 33.85 | trim adapters |
| JMS-12-2 | 3.83% | 37.89% | 30.50 | trim adapters |
| JMS-12-3 | 5.58% | 51.62% | 32.65 | trim adapters |
| JMS-12-4 | 6.82% | 60.13% | 34.28 | trim adapters |
| JMS-12-5 | 5.65% | 52.14% | 32.74 | trim adapters |
| JMS-12-6 | 3.85% | 34.85% | 33.35 | trim adapters |
| MS1-122-1 | 5.35% | 47.52% | 34.01 | trim adapters |
| MS1-122-2 | 3.95% | 35.16% | 33.94 | trim adapters |
| MS1-122-3 | 3.01% | 27.39% | 33.14 | trim adapters |
| MS1-122-4 | 3.73% | 33.25% | 33.85 | trim adapters |
| MS1-122-5 | 3.43% | 30.58% | 33.88 | trim adapters |
| MS1-122-6 | 4.29% | 39.96% | 32.39 | trim adapters |
| MS1-36-1 | 3.07% | 30.08% | 30.82 | trim adapters |
| MS1-36-2 | 4.01% | 37.71% | 32.15 | trim adapters |
| MS1-36-3 | 4.08% | 37.45% | 32.94 | trim adapters |
| MS1-36-4 | 6.61% | 59.95% | 33.32 | trim adapters |
| MS1-36-5 | 2.92% | 27.10% | 32.53 | trim adapters |
| MS1-36-6 | 3.61% | 31.80% | 34.29 | trim adapters |
| MS1-41-1 | 5.61% | 49.56% | 34.20 | trim adapters |
| MS1-41-2 | 4.68% | 41.18% | 34.29 | trim adapters |
| MS1-41-3 | 4.39% | 40.28% | 32.90 | trim adapters |
| MS1-41-4 | 4.24% | 37.93% | 33.72 | trim adapters |
| MS1-41-5 | 4.09% | 36.70% | 33.69 | trim adapters |
| MS1-41-6 | 4.01% | 35.78% | 33.82 | trim adapters |
| MS1-56-1 | 3.16% | 30.22% | 31.62 | trim adapters |
| MS1-56-2 | 3.65% | 34.03% | 32.41 | trim adapters |
| MS1-56-3 | 4.63% | 42.25% | 33.10 | trim adapters |
| MS1-56-4 | 5.11% | 48.52% | 31.79 | trim adapters |
| MS1-56-5 | 4.00% | 36.64% | 33.01 | trim adapters |
| MS1-56-6 | 4.09% | 38.09% | 32.40 | trim adapters |
| STJM-4-1 | 2.32% | 21.79% | 32.22 | trim adapters |
| STJM-4-2 | 3.28% | 29.96% | 33.09 | trim adapters |
| STJM-4-3 | 2.57% | 24.14% | 32.12 | trim adapters |
| STJM-4-4 | 3.58% | 32.58% | 33.16 | trim adapters |
| STJM-4-5 | 3.25% | 30.31% | 32.40 | trim adapters |
| STJM-4-6 | 4.05% | 37.18% | 32.86 | trim adapters |

×

#### Table Watj: Columns

Uncheck the tick box to hide columns. Click and drag the handle on the left to change order.

Show All
Show None

| Sort | Visible | Group | Column | Description | ID | Scale |
| --- | --- | --- | --- | --- | --- | --- |
| || |  | % Bp Lost | % Bp Lost | Percentage of Input bps (SE and PE) trimmed. | `At_%_BP_Lost1` | None |
| || |  | % Adapters | % Adapters | Percentage of Reads (SE and PE) with an Adapter | `At_%_Adapters1` | None |
| || |  | Avg. Bps Trimmed | Avg. Bps Trimmed | Average Number of basepairs trimmed from reads | `At_Avg_BP_Trimmed1` | None |
| || |  | Notes | Notes | Notes | `At_Notes1` | None |

Close

  

#### AdapterTrimmer: Trimmed Basepairs Composition

Composition of basepairs trimmed from the ends of paired end and single end reads.

Counts
Percentages

loading..

---

### hts\_PolyATTrim

Attempts to trim poly-A and poly-T sequences from the end of reads.

 Copy table

 Configure Columns

 Sort by highlight

 Plot
Showing 42/42 rows and 4/4 columns.

| Sample Name | Total Bp Lost | % R1 of Bp Lost | % R2 of Bp Lost | Notes |
| --- | --- | --- | --- | --- |
| 29JM-11-1 | 25189 | 0.00% | 100.00% | remove polyAT tails |
| 29JM-11-2 | 11035 | 0.00% | 100.00% | remove polyAT tails |
| 29JM-11-3 | 40364 | 0.00% | 100.00% | remove polyAT tails |
| 29JM-11-4 | 76357 | 0.00% | 100.00% | remove polyAT tails |
| 29JM-11-5 | 85171 | 0.00% | 100.00% | remove polyAT tails |
| 29JM-11-6 | 100950 | 0.00% | 100.00% | remove polyAT tails |
| JMS-12-1 | 67682 | 0.00% | 100.00% | remove polyAT tails |
| JMS-12-2 | 61972 | 0.00% | 100.00% | remove polyAT tails |
| JMS-12-3 | 84637 | 0.00% | 100.00% | remove polyAT tails |
| JMS-12-4 | 65092 | 0.00% | 100.00% | remove polyAT tails |
| JMS-12-5 | 88380 | 0.00% | 100.00% | remove polyAT tails |
| JMS-12-6 | 106881 | 0.00% | 100.00% | remove polyAT tails |
| MS1-122-1 | 56933 | 0.00% | 100.00% | remove polyAT tails |
| MS1-122-2 | 79320 | 0.00% | 100.00% | remove polyAT tails |
| MS1-122-3 | 90020 | 0.00% | 100.00% | remove polyAT tails |
| MS1-122-4 | 42163 | 0.00% | 100.00% | remove polyAT tails |
| MS1-122-5 | 120556 | 0.00% | 100.00% | remove polyAT tails |
| MS1-122-6 | 52842 | 0.00% | 100.00% | remove polyAT tails |
| MS1-36-1 | 112492 | 0.00% | 100.00% | remove polyAT tails |
| MS1-36-2 | 79717 | 0.00% | 100.00% | remove polyAT tails |
| MS1-36-3 | 191250 | 0.00% | 100.00% | remove polyAT tails |
| MS1-36-4 | 81856 | 0.00% | 100.00% | remove polyAT tails |
| MS1-36-5 | 46937 | 0.00% | 100.00% | remove polyAT tails |
| MS1-36-6 | 81022 | 0.00% | 100.00% | remove polyAT tails |
| MS1-41-1 | 64490 | 0.00% | 100.00% | remove polyAT tails |
| MS1-41-2 | 34000 | 0.00% | 100.00% | remove polyAT tails |
| MS1-41-3 | 57790 | 0.00% | 100.00% | remove polyAT tails |
| MS1-41-4 | 54140 | 0.00% | 100.00% | remove polyAT tails |
| MS1-41-5 | 63013 | 0.00% | 100.00% | remove polyAT tails |
| MS1-41-6 | 57626 | 0.00% | 100.00% | remove polyAT tails |
| MS1-56-1 | 90586 | 0.00% | 100.00% | remove polyAT tails |
| MS1-56-2 | 78266 | 0.00% | 100.00% | remove polyAT tails |
| MS1-56-3 | 85127 | 0.00% | 100.00% | remove polyAT tails |
| MS1-56-4 | 58196 | 0.00% | 100.00% | remove polyAT tails |
| MS1-56-5 | 20103 | 0.00% | 100.00% | remove polyAT tails |
| MS1-56-6 | 99142 | 0.00% | 100.00% | remove polyAT tails |
| STJM-4-1 | 7725 | 0.00% | 100.00% | remove polyAT tails |
| STJM-4-2 | 29193 | 0.00% | 100.00% | remove polyAT tails |
| STJM-4-3 | 20113 | 0.00% | 100.00% | remove polyAT tails |
| STJM-4-4 | 41296 | 0.00% | 100.00% | remove polyAT tails |
| STJM-4-5 | 18833 | 0.00% | 100.00% | remove polyAT tails |
| STJM-4-6 | 22744 | 0.00% | 100.00% | remove polyAT tails |

×

#### Table Igqz: Columns

Uncheck the tick box to hide columns. Click and drag the handle on the left to change order.

Show All
Show None

| Sort | Visible | Group | Column | Description | ID | Scale |
| --- | --- | --- | --- | --- | --- | --- |
| || |  | Total Bp Lost | Total Bp Lost | Total input bps (SE and PE) trimmed. | `Pt_BP_Lost1` | None |
| || |  | % Bp Lost from R1 | % R1 of Bp Lost | Percentage of total trimmed bps. | `Pt_%_R1_BP_Lost1` | None |
| || |  | % Bp Lost from R2 | % R2 of Bp Lost | Percentage of total trimmed bps. | `Pt_%_R2_BP_Lost1` | None |
| || |  | Notes | Notes | Notes | `Pt_Notes1` | None |

Close

---

### hts\_NTrimmer

Trims reads to the longest subsequence that contains no N's.

 Copy table

 Configure Columns

 Sort by highlight

 Plot
Showing 42/42 rows and 5/5 columns.

| Sample Name | Total Bp Lost | % R1 of Bp Lost | % R2 of Bp Lost | % Discarded | Notes |
| --- | --- | --- | --- | --- | --- |
| 29JM-11-1 | 0 | 0.00% | 0.00% | 0.00% | remove any remaining N characters |
| 29JM-11-2 | 0 | 0.00% | 0.00% | 0.00% | remove any remaining N characters |
| 29JM-11-3 | 0 | 0.00% | 0.00% | 0.00% | remove any remaining N characters |
| 29JM-11-4 | 0 | 0.00% | 0.00% | 0.00% | remove any remaining N characters |
| 29JM-11-5 | 0 | 0.00% | 0.00% | 0.00% | remove any remaining N characters |
| 29JM-11-6 | 0 | 0.00% | 0.00% | 0.00% | remove any remaining N characters |
| JMS-12-1 | 0 | 0.00% | 0.00% | 0.00% | remove any remaining N characters |
| JMS-12-2 | 0 | 0.00% | 0.00% | 0.00% | remove any remaining N characters |
| JMS-12-3 | 0 | 0.00% | 0.00% | 0.00% | remove any remaining N characters |
| JMS-12-4 | 0 | 0.00% | 0.00% | 0.00% | remove any remaining N characters |
| JMS-12-5 | 0 | 0.00% | 0.00% | 0.00% | remove any remaining N characters |
| JMS-12-6 | 0 | 0.00% | 0.00% | 0.00% | remove any remaining N characters |
| MS1-122-1 | 0 | 0.00% | 0.00% | 0.00% | remove any remaining N characters |
| MS1-122-2 | 0 | 0.00% | 0.00% | 0.00% | remove any remaining N characters |
| MS1-122-3 | 0 | 0.00% | 0.00% | 0.00% | remove any remaining N characters |
| MS1-122-4 | 0 | 0.00% | 0.00% | 0.00% | remove any remaining N characters |
| MS1-122-5 | 0 | 0.00% | 0.00% | 0.00% | remove any remaining N characters |
| MS1-122-6 | 0 | 0.00% | 0.00% | 0.00% | remove any remaining N characters |
| MS1-36-1 | 0 | 0.00% | 0.00% | 0.00% | remove any remaining N characters |
| MS1-36-2 | 0 | 0.00% | 0.00% | 0.00% | remove any remaining N characters |
| MS1-36-3 | 0 | 0.00% | 0.00% | 0.00% | remove any remaining N characters |
| MS1-36-4 | 0 | 0.00% | 0.00% | 0.00% | remove any remaining N characters |
| MS1-36-5 | 0 | 0.00% | 0.00% | 0.00% | remove any remaining N characters |
| MS1-36-6 | 0 | 0.00% | 0.00% | 0.00% | remove any remaining N characters |
| MS1-41-1 | 0 | 0.00% | 0.00% | 0.00% | remove any remaining N characters |
| MS1-41-2 | 0 | 0.00% | 0.00% | 0.00% | remove any remaining N characters |
| MS1-41-3 | 0 | 0.00% | 0.00% | 0.00% | remove any remaining N characters |
| MS1-41-4 | -1 | -0.00% | 100.00% | 0.00% | remove any remaining N characters |
| MS1-41-5 | 0 | 0.00% | 0.00% | 0.00% | remove any remaining N characters |
| MS1-41-6 | 0 | 0.00% | 0.00% | 0.00% | remove any remaining N characters |
| MS1-56-1 | 0 | 0.00% | 0.00% | 0.00% | remove any remaining N characters |
| MS1-56-2 | 0 | 0.00% | 0.00% | 0.00% | remove any remaining N characters |
| MS1-56-3 | 0 | 0.00% | 0.00% | 0.00% | remove any remaining N characters |
| MS1-56-4 | 0 | 0.00% | 0.00% | 0.00% | remove any remaining N characters |
| MS1-56-5 | 0 | 0.00% | 0.00% | 0.00% | remove any remaining N characters |
| MS1-56-6 | 0 | 0.00% | 0.00% | 0.00% | remove any remaining N characters |
| STJM-4-1 | 0 | 0.00% | 0.00% | 0.00% | remove any remaining N characters |
| STJM-4-2 | 0 | 0.00% | 0.00% | 0.00% | remove any remaining N characters |
| STJM-4-3 | 0 | 0.00% | 0.00% | 0.00% | remove any remaining N characters |
| STJM-4-4 | 0 | 0.00% | 0.00% | 0.00% | remove any remaining N characters |
| STJM-4-5 | 0 | 0.00% | 0.00% | 0.00% | remove any remaining N characters |
| STJM-4-6 | 0 | 0.00% | 0.00% | 0.00% | remove any remaining N characters |

×

#### Table Owdv: Columns

Uncheck the tick box to hide columns. Click and drag the handle on the left to change order.

Show All
Show None

| Sort | Visible | Group | Column | Description | ID | Scale |
| --- | --- | --- | --- | --- | --- | --- |
| || |  | Total Bp Lost | Total Bp Lost | Total input bps (SE and PE) trimmed. | `Nt_BP_Lost1` | None |
| || |  | % Bp Lost from R1 | % R1 of Bp Lost | Percentage of total trimmed bps. | `Nt_%_R1_BP_Lost1` | None |
| || |  | % Bp Lost from R2 | % R2 of Bp Lost | Percentage of total trimmed bps. | `Nt_%_R2_BP_Lost1` | None |
| || |  | % Discarded | % Discarded | Percentage of Reads (SE and PE) Discarded | `Nt_%_Discarded1` | None |
| || |  | Notes | Notes | Notes | `Nt_Notes1` | None |

Close

  

#### NTrimmer: Trimmed Basepairs Composition

Plots the number of N bases trimmed from ends of paired end and single end reads.

Counts
Percentages

loading..

---

### hts\_QWindowTrim

Uses a sliding window approach to remove the low quality ends of reads.

 Copy table

 Configure Columns

 Sort by highlight

 Plot
Showing 42/42 rows and 5/5 columns.

| Sample Name | % Bp Lost | % R1 of Bp Lost | % R2 of Bp Lost | Avg. Bps Trimmed | Notes |
| --- | --- | --- | --- | --- | --- |
| 29JM-11-1 | 0.48% | 18.72% | 81.28% | 1.38 | quality trim the ends of reads |
| 29JM-11-2 | 0.69% | 18.70% | 81.30% | 2.03 | quality trim the ends of reads |
| 29JM-11-3 | 0.33% | 23.14% | 76.86% | 0.94 | quality trim the ends of reads |
| 29JM-11-4 | 0.22% | 31.36% | 68.64% | 0.63 | quality trim the ends of reads |
| 29JM-11-5 | 0.24% | 28.38% | 71.62% | 0.69 | quality trim the ends of reads |
| 29JM-11-6 | 0.23% | 30.91% | 69.09% | 0.65 | quality trim the ends of reads |
| JMS-12-1 | 0.25% | 26.21% | 73.79% | 0.72 | quality trim the ends of reads |
| JMS-12-2 | 0.27% | 26.39% | 73.61% | 0.78 | quality trim the ends of reads |
| JMS-12-3 | 0.24% | 28.49% | 71.51% | 0.70 | quality trim the ends of reads |
| JMS-12-4 | 0.24% | 27.58% | 72.42% | 0.69 | quality trim the ends of reads |
| JMS-12-5 | 0.25% | 27.58% | 72.42% | 0.72 | quality trim the ends of reads |
| JMS-12-6 | 0.20% | 34.01% | 65.99% | 0.57 | quality trim the ends of reads |
| MS1-122-1 | 0.33% | 21.48% | 78.52% | 0.94 | quality trim the ends of reads |
| MS1-122-2 | 0.20% | 32.66% | 67.34% | 0.58 | quality trim the ends of reads |
| MS1-122-3 | 0.20% | 31.14% | 68.86% | 0.60 | quality trim the ends of reads |
| MS1-122-4 | 0.28% | 27.82% | 72.18% | 0.83 | quality trim the ends of reads |
| MS1-122-5 | 0.19% | 34.34% | 65.66% | 0.54 | quality trim the ends of reads |
| MS1-122-6 | 0.28% | 30.06% | 69.94% | 0.81 | quality trim the ends of reads |
| MS1-36-1 | 0.24% | 30.07% | 69.93% | 0.70 | quality trim the ends of reads |
| MS1-36-2 | 0.32% | 28.01% | 71.99% | 0.93 | quality trim the ends of reads |
| MS1-36-3 | 0.22% | 31.88% | 68.12% | 0.63 | quality trim the ends of reads |
| MS1-36-4 | 0.24% | 25.23% | 74.77% | 0.69 | quality trim the ends of reads |
| MS1-36-5 | 0.25% | 31.41% | 68.59% | 0.73 | quality trim the ends of reads |
| MS1-36-6 | 0.29% | 26.46% | 73.54% | 0.85 | quality trim the ends of reads |
| MS1-41-1 | 0.25% | 31.08% | 68.92% | 0.72 | quality trim the ends of reads |
| MS1-41-2 | 0.32% | 24.78% | 75.22% | 0.93 | quality trim the ends of reads |
| MS1-41-3 | 0.27% | 25.50% | 74.50% | 0.79 | quality trim the ends of reads |
| MS1-41-4 | 0.25% | 26.88% | 73.12% | 0.72 | quality trim the ends of reads |
| MS1-41-5 | 0.27% | 28.21% | 71.79% | 0.79 | quality trim the ends of reads |
| MS1-41-6 | 0.27% | 27.84% | 72.16% | 0.79 | quality trim the ends of reads |
| MS1-56-1 | 0.27% | 30.69% | 69.31% | 0.78 | quality trim the ends of reads |
| MS1-56-2 | 0.25% | 32.78% | 67.22% | 0.71 | quality trim the ends of reads |
| MS1-56-3 | 0.23% | 30.84% | 69.16% | 0.67 | quality trim the ends of reads |
| MS1-56-4 | 0.24% | 27.01% | 72.99% | 0.68 | quality trim the ends of reads |
| MS1-56-5 | 0.38% | 27.69% | 72.31% | 1.11 | quality trim the ends of reads |
| MS1-56-6 | 0.25% | 31.93% | 68.07% | 0.74 | quality trim the ends of reads |
| STJM-4-1 | 0.65% | 22.38% | 77.62% | 1.91 | quality trim the ends of reads |
| STJM-4-2 | 0.32% | 29.61% | 70.39% | 0.93 | quality trim the ends of reads |
| STJM-4-3 | 0.53% | 18.56% | 81.44% | 1.55 | quality trim the ends of reads |
| STJM-4-4 | 0.26% | 27.98% | 72.02% | 0.77 | quality trim the ends of reads |
| STJM-4-5 | 0.52% | 21.66% | 78.34% | 1.53 | quality trim the ends of reads |
| STJM-4-6 | 0.48% | 19.27% | 80.73% | 1.38 | quality trim the ends of reads |

×

#### Table Uyiq: Columns

Uncheck the tick box to hide columns. Click and drag the handle on the left to change order.

Show All
Show None

| Sort | Visible | Group | Column | Description | ID | Scale |
| --- | --- | --- | --- | --- | --- | --- |
| || |  | % Bp Lost | % Bp Lost | Percentage of Input bps (SE and PE) trimmed. | `Qt_%_BP_Lost1` | None |
| || |  | % Bp Lost from R1 | % R1 of Bp Lost | Percentage of total trimmed bps. | `Qt_%_R1_BP_Lost1` | None |
| || |  | % Bp Lost from R2 | % R2 of Bp Lost | Percentage of total trimmed bps. | `Qt_%_R2_BP_Lost1` | None |
| || |  | Avg. Bpss Trimmed | Avg. Bps Trimmed | Average Number of Basepairs Trimmed per Read | `Qt_Avg_BP_Trimmed1` | None |
| || |  | Notes | Notes | Notes | `Qt_Notes1` | None |

Close

  

#### QWindowTrim: Trimmed Basepairs Composition

Plots the number of low quality basepairs trimmed from ends of paired end and single end reads.

Counts
Percentages

Read 1
Read 2

loading..

---

### hts\_LengthFilter

Discards reads below a minimum length threshold.

 Copy table

 Configure Columns

 Sort by highlight

 Plot
Showing 42/42 rows and 2/2 columns.

| Sample Name | % PE Lost | Notes |
| --- | --- | --- |
| 29JM-11-1 | 0.13% | remove reads < 50bp |
| 29JM-11-2 | 0.26% | remove reads < 50bp |
| 29JM-11-3 | 0.09% | remove reads < 50bp |
| 29JM-11-4 | 0.04% | remove reads < 50bp |
| 29JM-11-5 | 0.06% | remove reads < 50bp |
| 29JM-11-6 | 0.04% | remove reads < 50bp |
| JMS-12-1 | 0.06% | remove reads < 50bp |
| JMS-12-2 | 0.07% | remove reads < 50bp |
| JMS-12-3 | 0.05% | remove reads < 50bp |
| JMS-12-4 | 0.05% | remove reads < 50bp |
| JMS-12-5 | 0.06% | remove reads < 50bp |
| JMS-12-6 | 0.03% | remove reads < 50bp |
| MS1-122-1 | 0.12% | remove reads < 50bp |
| MS1-122-2 | 0.04% | remove reads < 50bp |
| MS1-122-3 | 0.04% | remove reads < 50bp |
| MS1-122-4 | 0.05% | remove reads < 50bp |
| MS1-122-5 | 0.03% | remove reads < 50bp |
| MS1-122-6 | 0.05% | remove reads < 50bp |
| MS1-36-1 | 0.05% | remove reads < 50bp |
| MS1-36-2 | 0.06% | remove reads < 50bp |
| MS1-36-3 | 0.04% | remove reads < 50bp |
| MS1-36-4 | 0.07% | remove reads < 50bp |
| MS1-36-5 | 0.05% | remove reads < 50bp |
| MS1-36-6 | 0.06% | remove reads < 50bp |
| MS1-41-1 | 0.05% | remove reads < 50bp |
| MS1-41-2 | 0.08% | remove reads < 50bp |
| MS1-41-3 | 0.07% | remove reads < 50bp |
| MS1-41-4 | 0.07% | remove reads < 50bp |
| MS1-41-5 | 0.05% | remove reads < 50bp |
| MS1-41-6 | 0.06% | remove reads < 50bp |
| MS1-56-1 | 0.05% | remove reads < 50bp |
| MS1-56-2 | 0.04% | remove reads < 50bp |
| MS1-56-3 | 0.05% | remove reads < 50bp |
| MS1-56-4 | 0.06% | remove reads < 50bp |
| MS1-56-5 | 0.06% | remove reads < 50bp |
| MS1-56-6 | 0.04% | remove reads < 50bp |
| STJM-4-1 | 0.15% | remove reads < 50bp |
| STJM-4-2 | 0.06% | remove reads < 50bp |
| STJM-4-3 | 0.18% | remove reads < 50bp |
| STJM-4-4 | 0.05% | remove reads < 50bp |
| STJM-4-5 | 0.17% | remove reads < 50bp |
| STJM-4-6 | 0.14% | remove reads < 50bp |

×

#### Table Pzuj: Columns

Uncheck the tick box to hide columns. Click and drag the handle on the left to change order.

Show All
Show None

| Sort | Visible | Group | Column | Description | ID | Scale |
| --- | --- | --- | --- | --- | --- | --- |
| || |  | % PE Lost | % PE Lost | Percentage of Paired End Reads Lost | `Lf_PE_loss1` | None |
| || |  | Notes | Notes | Notes | `Lf_Notes1` | None |

Close

---

### hts\_Stats 2

Generates a JSON formatted file containing a set of statistical measures about the input read data.

 Copy table

 Configure Columns

 Sort by highlight

 Plot
Showing 42/42 rows and 6/6 columns.

| Sample Name | % PE | % R1 Q30 | % R2 Q30 | GC Content | N Content | Notes |
| --- | --- | --- | --- | --- | --- | --- |
| 29JM-11-1 | 100.00% | 93.68% | 88.40% | 47.82% | 0.0000% | final stats |
| 29JM-11-2 | 100.00% | 90.32% | 84.23% | 48.40% | 0.0000% | final stats |
| 29JM-11-3 | 100.00% | 94.22% | 90.62% | 46.45% | 0.0000% | final stats |
| 29JM-11-4 | 100.00% | 94.65% | 91.86% | 46.20% | 0.0000% | final stats |
| 29JM-11-5 | 100.00% | 94.73% | 91.86% | 46.31% | 0.0000% | final stats |
| 29JM-11-6 | 100.00% | 95.01% | 91.84% | 46.48% | 0.0000% | final stats |
| JMS-12-1 | 100.00% | 94.99% | 91.86% | 45.83% | 0.0000% | final stats |
| JMS-12-2 | 100.00% | 94.83% | 91.57% | 46.36% | 0.0000% | final stats |
| JMS-12-3 | 100.00% | 95.16% | 91.95% | 46.01% | 0.0000% | final stats |
| JMS-12-4 | 100.00% | 95.35% | 92.19% | 45.74% | 0.0000% | final stats |
| JMS-12-5 | 100.00% | 95.06% | 92.00% | 45.77% | 0.0000% | final stats |
| JMS-12-6 | 100.00% | 94.68% | 91.91% | 45.85% | 0.0000% | final stats |
| MS1-122-1 | 100.00% | 94.77% | 90.76% | 45.34% | 0.0000% | final stats |
| MS1-122-2 | 100.00% | 94.45% | 91.89% | 45.83% | 0.0000% | final stats |
| MS1-122-3 | 100.00% | 94.22% | 90.27% | 45.95% | 0.0000% | final stats |
| MS1-122-4 | 100.00% | 93.46% | 89.40% | 47.12% | 0.0000% | final stats |
| MS1-122-5 | 100.00% | 94.19% | 91.57% | 45.65% | 0.0000% | final stats |
| MS1-122-6 | 100.00% | 94.13% | 90.67% | 46.96% | 0.0000% | final stats |
| MS1-36-1 | 100.00% | 94.38% | 90.77% | 46.02% | 0.0000% | final stats |
| MS1-36-2 | 100.00% | 93.73% | 88.40% | 46.49% | 0.0000% | final stats |
| MS1-36-3 | 100.00% | 94.72% | 90.91% | 45.28% | 0.0000% | final stats |
| MS1-36-4 | 100.00% | 95.72% | 92.84% | 45.43% | 0.0000% | final stats |
| MS1-36-5 | 100.00% | 93.82% | 89.88% | 46.52% | 0.0000% | final stats |
| MS1-36-6 | 100.00% | 93.91% | 89.79% | 46.32% | 0.0000% | final stats |
| MS1-41-1 | 100.00% | 94.78% | 91.22% | 46.08% | 0.0000% | final stats |
| MS1-41-2 | 100.00% | 94.07% | 90.30% | 47.47% | 0.0000% | final stats |
| MS1-41-3 | 100.00% | 94.68% | 91.18% | 46.14% | 0.0000% | final stats |
| MS1-41-4 | 100.00% | 94.50% | 91.29% | 46.55% | 0.0000% | final stats |
| MS1-41-5 | 100.00% | 94.10% | 90.61% | 47.18% | 0.0000% | final stats |
| MS1-41-6 | 100.00% | 94.19% | 90.69% | 46.38% | 0.0000% | final stats |
| MS1-56-1 | 100.00% | 94.05% | 90.70% | 46.04% | 0.0000% | final stats |
| MS1-56-2 | 100.00% | 94.17% | 90.72% | 46.51% | 0.0000% | final stats |
| MS1-56-3 | 100.00% | 94.70% | 91.80% | 46.41% | 0.0000% | final stats |
| MS1-56-4 | 100.00% | 95.36% | 92.56% | 46.50% | 0.0000% | final stats |
| MS1-56-5 | 100.00% | 92.66% | 88.84% | 49.44% | 0.0000% | final stats |
| MS1-56-6 | 100.00% | 94.45% | 91.09% | 46.94% | 0.0000% | final stats |
| STJM-4-1 | 100.00% | 90.04% | 84.28% | 50.21% | 0.0000% | final stats |
| STJM-4-2 | 100.00% | 93.27% | 89.48% | 47.37% | 0.0000% | final stats |
| STJM-4-3 | 100.00% | 92.42% | 87.08% | 46.87% | 0.0000% | final stats |
| STJM-4-4 | 100.00% | 94.28% | 90.76% | 47.31% | 0.0000% | final stats |
| STJM-4-5 | 100.00% | 91.91% | 86.87% | 47.89% | 0.0000% | final stats |
| STJM-4-6 | 100.00% | 93.30% | 88.29% | 47.82% | 0.0000% | final stats |

×

#### Table Jrul: Columns

Uncheck the tick box to hide columns. Click and drag the handle on the left to change order.

Show All
Show None

| Sort | Visible | Group | Column | Description | ID | Scale |
| --- | --- | --- | --- | --- | --- | --- |
| || |  | % PE | % PE | percentage of paried end reads | `St_PE_Fraction2` | None |
| || |  | % R1 Q30 | % R1 Q30 | percentage of read 1 bps Q30 or greater | `St_R1_Q302` | None |
| || |  | % R2 Q30 | % R2 Q30 | percentage of read 2 bps Q30 or greater | `St_R2_Q302` | None |
| || |  | GC Content | GC Content | Percentage of bps that are G or C | `St_GC_Content2` | None |
| || |  | N Content | N Content | Percentage of bps that are N | `St_N_Content2` | None |
| || |  | Notes | Notes | Notes | `St_Notes2` | None |

Close

  

#### Read Lengths: Paired End

Distribution of read lengths for each sample.

Sort by highlight

loading..

  

#### Base by Cycle: Paired End

Provides a measure of the uniformity of a distribution. The higher the average is at a certain position,
the more unequal the base pair composition. N's are excluded from this calculation.

Avg. Diff. from 25%
Base by Cycle

loading..

29JM-11-1
29JM-11-2
29JM-11-3
29JM-11-4
29JM-11-5
29JM-11-6
JMS-12-1
JMS-12-2
JMS-12-3
JMS-12-4
JMS-12-5
JMS-12-6
MS1-122-1
MS1-122-2
MS1-122-3
MS1-122-4
MS1-122-5
MS1-122-6
MS1-36-1
MS1-36-2
MS1-36-3
MS1-36-4
MS1-36-5
MS1-36-6
MS1-41-1
MS1-41-2
MS1-41-3
MS1-41-4
MS1-41-5
MS1-41-6
MS1-56-1
MS1-56-2
MS1-56-3
MS1-56-4
MS1-56-5
MS1-56-6
STJM-4-1
STJM-4-2
STJM-4-3
STJM-4-4
STJM-4-5
STJM-4-6

loading..

  

#### Quality by Cycle: Paired End

Mean quality score for each position along the read.
Sample is colored red if less than 60% of bps have mean score of at least Q30,
orange if between 60% and 80%, and green otherwise.

Mean Quality
Quality by Cycle

loading..

29JM-11-1
29JM-11-2
29JM-11-3
29JM-11-4
29JM-11-5
29JM-11-6
JMS-12-1
JMS-12-2
JMS-12-3
JMS-12-4
JMS-12-5
JMS-12-6
MS1-122-1
MS1-122-2
MS1-122-3
MS1-122-4
MS1-122-5
MS1-122-6
MS1-36-1
MS1-36-2
MS1-36-3
MS1-36-4
MS1-36-5
MS1-36-6
MS1-41-1
MS1-41-2
MS1-41-3
MS1-41-4
MS1-41-5
MS1-41-6
MS1-56-1
MS1-56-2
MS1-56-3
MS1-56-4
MS1-56-5
MS1-56-6
STJM-4-1
STJM-4-2
STJM-4-3
STJM-4-4
STJM-4-5
STJM-4-6

Sort by highlight

loading..

**MultiQC v1.10.dev0**
- Written by Phil Ewels,
available on GitHub.

This report uses HighCharts,
jQuery,
jQuery UI,
Bootstrap,
FileSaver.js and
clipboard.js.

×

### Plot Table Data

Select Column

Select Column

Please select two table columns.

Close

×

### Regex Help

Toolbox search strings can behave as regular expressions (regexes). Click a button below to see an example of it in action. Try modifying them yourself in the text box.

`^` (start of string)
`$` (end of string)
`[]` (character choice)
`\d` (shorthand for `[0-9]`)
`\w` (shorthand for `[0-9a-zA-Z_]`)
`.` (any character)
`\.` (literal full stop)
`()` `|` (group / separator)
`*` (prev char 0 or more)
`+` (prev char 1 or more)
`?` (prev char 0 or 1)
`{}` (char num times)
`{,}` (count range)

```
samp_1
samp_1_edited
samp_2
samp_2_edited
samp_3
samp_3_edited
prepended_samp_1
tmp_samp_1_edited
tmpp_samp_1_edited
tmppp_samp_1_edited
#samp_1_edited.tmp
samp_11
samp_11111
```

See regex101.com for a more heavy duty testing suite.

Close
