# Supplementary figures and images for: Transcriptomic Evidence of a Link between Cell Wall Biogenesis, Pathogenesis, and Vigor in Walnut Root and Trunk Diseases
Source: Int J Mol Sci. 2024 Jan 11;25(2):931. doi: 10.3390/ijms25020931 (PMC10815794; doi:10.3390/ijms25020931)

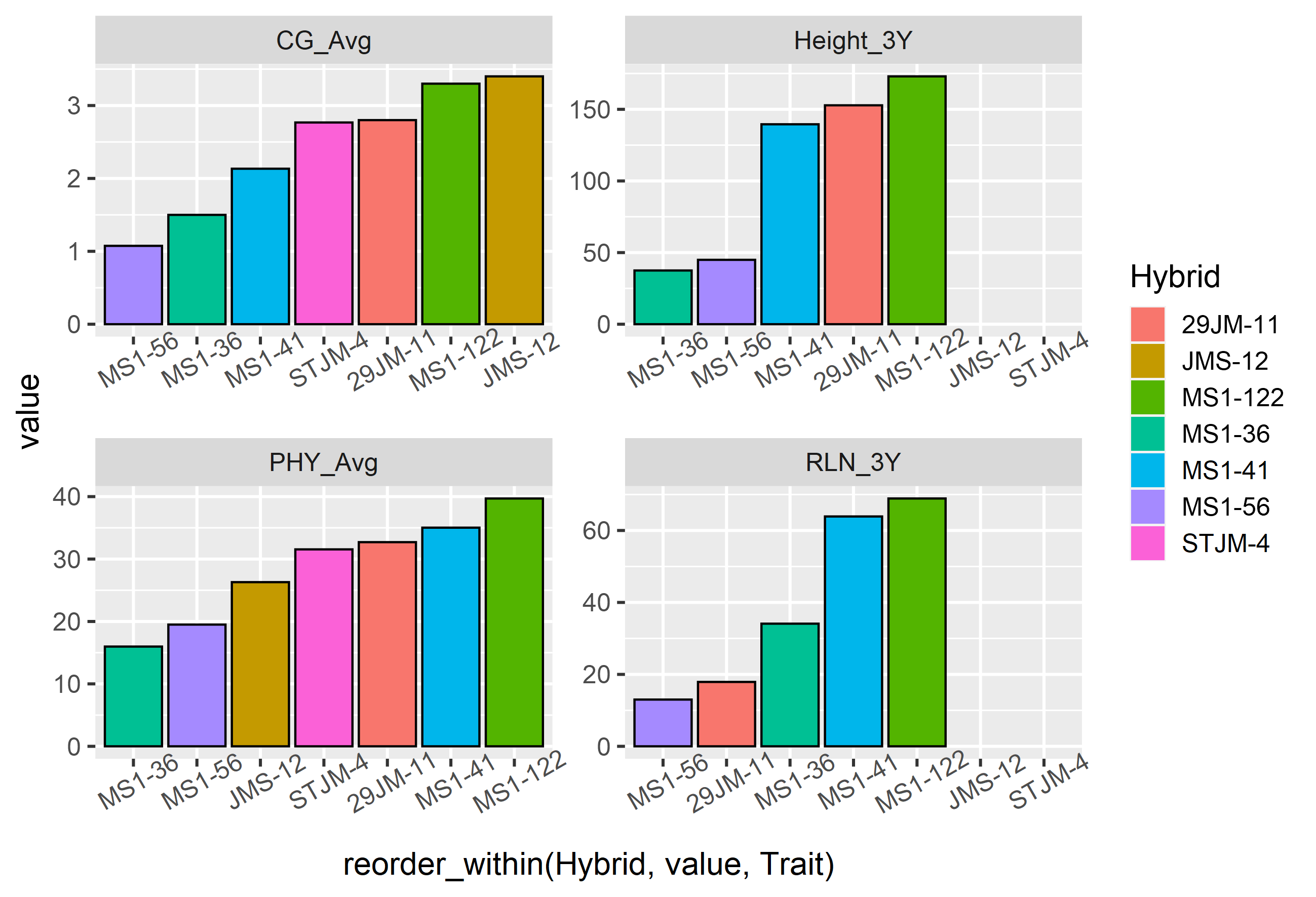

Supplement: Supplementary file 1 [file ijms-25-00931-s001.zip › Fig. S1.png]

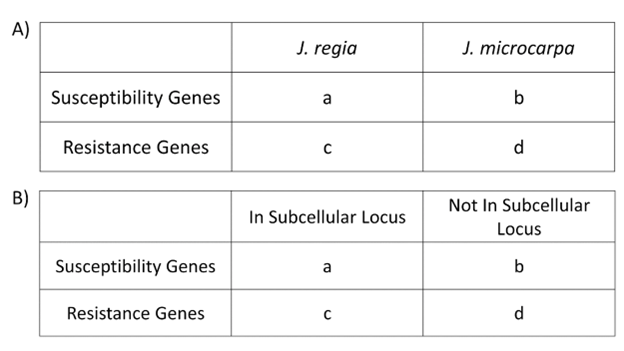

Supplement: Supplementary file 1 [file ijms-25-00931-s001.zip › Fig. S2.png]
